# Evidence of Antitumor and Antimetastatic Potential of Induced Pluripotent Stem Cell-Based Vaccines in Cancer Immunotherapy

**DOI:** 10.3389/fmed.2021.729018

**Published:** 2021-12-10

**Authors:** Masae Kishi, Afag Asgarova, Christophe Desterke, Diana Chaker, Jérôme Artus, Ali G. Turhan, Annelise Bennaceur-Griscelli, Frank Griscelli

**Affiliations:** ^1^Institut National de la Santé et de la Recherche Médicale (INSERM) UA9-Human Pluripotent Stem Cell Core Facility, CITHERA Infrastructure-INGESTEM, Villejuif, France; ^2^Université Paris-Saclay, Faculté de Médecine, Kremlin Bicêtre, France; ^3^APHP Paris-Saclay Service d'Hématologie, Hôpital Universitaire Paris Sud (AP-HP), Kremlin Bicêtre, France; ^4^Université Paris Descartes, Sorbonne Paris Cité, Faculté des Sciences Pharmaceutiques et Biologiques, Paris, France; ^5^Département de Biologie Médicale et Pathologie Médicales, Gustave Roussy Cancer Campus, Villejuif, France

**Keywords:** breast cancer, CSC, vaccine, iPSCs, HDACi

## Abstract

Cancer is maintained by the activity of a rare population of self-renewing “cancer stem cells” (CSCs), which are resistant to conventional therapies. CSCs over-express several proteins shared with induced pluripotent stem cells (iPSCs). We show here that allogenic or autologous murine iPSCs, combined with a histone deacetylase inhibitor (HDACi), are able to elicit major anti-tumor responses in a highly aggressive triple-negative breast cancer, as a relevant cancer stemness model. This immunotherapy strategy was effective in preventing tumor establishment and efficiently targeted CSCs by inducing extensive modifications of the tumor microenvironment. The anti-tumoral effect was correlated with the generation of CD4+, CD8+ T cells, and CD44+ CD62L- CCR7low CD127low T-effector memory cells, and the reduction of CD4+ CD25+FoxP3+ Tregs, Arg1^+^ CD11b+ Gr1+, and Arg1^+^ and CD11b+ Ly6+ myeloid-derived suppressor cell populations within the tumor. The anti-tumoral effect was associated with a reduction in metastatic dissemination and an improvement in the survival rate. These results demonstrate for the first time the clinical relevance of using an off-the-shelf allogeneic iPSC-based vaccine combined with an HDACi as a novel pan-cancer anti-cancer immunotherapy strategy against aggressive tumors harboring stemness features with high metastatic potential.

## Introduction

During the last decade, the concept of tumor heterogeneity has been extensively explored in solid tumors, leading to the identification, in several types of cancers, of dedifferentiated cancer cells, designated as “tumor-initiating cells” or “cancer stem cells” (CSCs) (1, 2). Within the bulk of a tumor, these cells represent a minor population with defined functional and molecular characteristics: (i) they are able to reinitiate tumor growth in immunodeficient mice by self-renewal capacity (3, 4), (ii) they exhibit various degrees of stemness signature based on transcriptomic and epigenetic molecular profile (5–7). Specifically, current evidence indicates that in addition to the well-known oncoprotein c-MYC, some of the key regulators of embryonic stem cells (ESCs), such as OCT4, SOX2, and NANOG, are also abnormally over-expressed in CSCs of a broad range of malignancies (7, 8). These three factors participate in a highly integrated network along with c-MYC and polycomb proteins that use the epigenetic machinery to remodel chromatin through histone modification and DNA methylation. This ability to induce major epigenetic modifications was first demonstrated by groundbreaking experiments leading to the discovery of induced pluripotent stem cell (iPSC) technology (9, 10). iPSCs are closely linked to ESCs, as both express the same auto-regulatory circuitries (11). Subsequent analyses of the genomic characteristics of iPSCs revealed the occurrence of genetic abnormalities in iPSC arising either from the initial somatic parental cell (12, 13) or during their expansion *in vitro* (12).

Previous studies have explored ESC/iPSC-based cancer vaccines as source of immunogenic tumor associated antigens (TAA) (14–18). However, they could not assess their effect on metastatic spread because the model cell line used in these studies lacked metastatic potential. Given these considerations, we used a cancer stemness model to explore an iPSC-based vaccination strategy exploring the possibility to inhibit metastatic dissemination. To this purpose, we have used an aggressive murine cancer cell line generating lung metastasis after implantation. In addition, as cancer stemness is strongly associated with immunosuppressive genes that inhibit T cell activation, we have combined the iPSC vaccination along with an epigenetic modification using HDAC inhibitors. It is indeed known that tumor microenvironment (TME) represents a privileged niche in which diversification of tumor clones can occur (19). It is also well-documented that this immunosuppressive niche contributes to the establishment, progression, and immune escape in various types of cancers. This niche is generated through epigenetic alterations capable of efficiently and accurately reprogramming the TME. The latter was shown to implicate several mechanisms including DNA methylation, histone post-translational modifications, and non-coding RNA-mediated regulation (20). Molecules such as histone deacetylase inhibitors (HDACi) are currently under evaluation to modify this immunosuppressive TME able to convert a tumor from an immune suppressive (cold) to an immune permissive (hot) niche (21). In a therapeutic point of view, the short-chain fatty antiepileptic drug Valproic Acid (VPA) is a class I selective HDACi, acting with IC50 values ranging from 0.4 to 3 mM, was shown to be able to modify TME by decreasing in particular myeloid-derived suppressor cells (MDSCs) (22) without significant side effects (23).

We show here that iPSCs-based vaccine combined with VPA in a metastatic model of aggressive murine cancer cell line, prevent the establishment of CSCs-enriched tumors and to inhibit efficiently the development of lung metastases.

## Materials and Methods

### Cell Line Isolation and Maintenance

Primary fibroblasts from BALB/c mice were reprogrammed into pluripotency by ectopic expression of OCT4, SOX2, c-MYC, and KLF4 using a Cre-Excisable Constitutive Polycistronic Lentivirus (EF1alpha-STEMCCA-LoxP backbone from Millipore). A C57BL/6-derived iPS cell line was purchased from ALSTEM (Richmond, CA). Both murine iPSC lines were maintained on mitomycin-treated MEFs in DMEM glutamax (Gibco), 15% fetal bovine serum (Eurobio), 1% penicillin and streptomycin (Invitrogen), 1 mM 2-mercaptoethanol (Sigma), and 1,000 units/mL of Leukemia Inhibitory Factor. Pluripotency was confirmed by FACS analysis and Teratoma assays in immunodeficient mice (NOD-SCID) after injection of 2 × 10^6^ iPSC per mouse. Two months later, the presence of three germline layers were confirmed in teratomas by histology and Immunohistochemistry (IHC) as described (24). The vaccine batch was prepared from miPSC cultured on gelatin (Sigma) and Essential 8 medium (Thermo Fisher Scientific). miPSCs were incubated for 24 h with 0.5 mM of VPA, known to improve the induction of bona fide iPSCs and to avoid iPSCs senescence (25, 26) followed by a lethal irradiation at 15 Gy. The breast cancer line 4T1 was obtained from ATCC (CRL-2539). 4T1 MammoSpheres (MSs) were produced in 9 days in low-attachment 6-well-plates at density of 100,000 cells per well in MEF-conditioned medium (3/4 MEF-conditioned medium + 1/4 mES medium + 4 ng/mL bFGF), and addition of TNF-alpha (20 ng/mL), and TGF-β 1 (10 ng/mL) (Cell Signaling Technology). 4T1 cell line was transduced by the retroviral vector pMEGIX encoding the genes for firefly luciferase. Stable clones of 4T-luc were isolated and selected by bioluminescence imaging systems based on luciferase expression.

### Transcriptome Meta-Analysis of 4T1 Cells and miPSCs

To confirm the stemness signature of murine 4T1, we have performed transcriptome microarray analysis in context of *in vitro* and *in vivo* tumor experiments (Clariom™ S Assay, mouse kit, Thermofisher scientific France) according to the manufacturer's instructions. Our 4T1 *in vitro* and 4T1 *in vivo* transplant transcriptome experiments were integrated with mammary gland samples from GEO dataset GSE14202 and mouse iPSC data from GEO dataset GSE15267. Cross batch normalization was applied with Combat function from SVA R bioconductor package (27). LIMMA analysis was performed between 4T1-transplant samples vs. in 4T1 *in vitro* samples to identify differential expressed genes (28). Expression heatmap was realized with pheatmap R-package and unsupervised principal component analysis plot ggfortify R package post prcomp R function. Functional enrichment was performed with Toppgene web application (29) on GO-BP, MSigDb and DisGeNET (30) databases. Functional enrichment network was built with Cytoscape application version 3.6.0 (31).

### Transcriptome Analysis of 4T1 Cells Treated With Valproic Acid

Total RNA was extracted following the instructions of the manufacturer (TRIzol, Life Technologies) from 4T1 cells treated with and without valproic acid (VPA) at dose of 0.5 mM for 10 days. Microarray probes were synthetized in one cycle of RNA amplification in which molecules were labeled (Affymetrix microarray station, Affymetrix, CA). The labeled microarray probes were hybridized on a Mouse Clariom S (mm10) microarray (Thermo Fisher Scientific), and the CEL files of microarray data obtained from the Affymetrix platform were normalized using the RMA method in Affymetrix Expression Console software (Affymetrix, CA). Gene-set enrichment analysis was performed with the online java module of GSEA software, version 3.0, while a network-based gene-set enrichment analysis was performed with Cytoscape software, version 3.6.0. Bioinformatics analyses were performed in R version 3.4.1; the R-package made4 was used to create an expression heatmap using Euclidean distances and the Ward method, and the FactoMineR R-package was used to perform an unsupervised principal component analysis. Genes with significant differences in expression were selected with the SAM algorithm using a FDR threshold of 5 percent.

### Animal Model

Wild-type female BALB/c mice, 8–10 weeks old, were purchased from Janvier Laboratory. Protocols of animal experiments were approved by the Animal Care Committee of the Val de Marne. *In vivo* studies were designed using 4 to 10 BALB/c mice in each group. Treatment consisted of two sub-cutaneous injections (1-week interval between injections) of 2 × 10^6^ miPSCs. One week following the second injection, mice were inoculated with 5 × 10^4^ 4T1Luc cells, directly transplanted into mammary fat-pad following by 15 days of VPA treatment, orally administered at dose of 4 mg/mL, an approach that leads to approximate concentrations of 0.4 mM VPA in plasma, as previously reported (32). Tumor growth was followed *in vivo* by bioluminescence imaging using the IVIS Spectrum system and quantified by the Living Image software (Perkin Elmer). Lungs were incubated *in vitro* with 150 μg/mL luciferin to quantify metastatic tumors. Tumors size and volume was measured and dissociated by GentleMACS dissociator (Miltenyi Biotec) without enzyme R (Tumor dissociation kit, Miltenyi Biotec). Spleens were dissociated using a cell strainer (Fisher), after removing red blood cells (RBC Lysis Buffer, eBioscience).

### Staining of Immune Cells and Tumor Cells for FACS Analysis

Splenocyte and immune cells from tumor micro-environment were analyzed by FACS for the expression of CD44, CD24, CD45, CD8a, CD25, CD279 (PD-1), MHC class I, CD3, CD11b, Gr-1, CD4, Ly-6C, CXCR5, CD22, CD62L, Arginase 1/ARG1, CD197, CD127 (Supplementary Table 1). Dead cells were excluded using 7-aminoactinomycin D (7-AAD) and zombie violet. Immune stimulation was performed using 50 ng/mL Phorbol 12-Myristate 13-Acetate (PMA) (Sigma) and 500 ng/mL ionomycin b (Sigma) in RPMI 1640 medium (Gibco). FACS analysis was conducted using MACSQuant analyzer (Miltenyi Biotec). The proportion of T-cell subpopulations has been reported among total CD3 + lymphocytes in analysis of blood samples. The proportion of cells in tumor samples and spleen, was reported among the total CD45 + hematopoietic cell population.

### Aldefluor Assay

The Aldefluor kit (Stem Cell Technologies) was used to characterize the ALDH activity on 4T1 cells as described (33). Cells were incubated at 37°C in Aldefluor assay buffer, which contained the ALDH substrate BODIPY-aminoacetaldehyde. To determine baseline fluorescence, the enzymatic activity of ALDH was blocked by the inhibitor DEAB. FACS analysis was performed using a MACSQuant analyzer (Miltenyi Biotec).

### Transcriptome Analysis of 4T1 Tumors From Mice Vaccinated With miPSCs and VPA

Total RNA was extracted from 4T1 tumors in treated and control mice, following the instructions of the manufacturer (TRIzol, Life Technologies). Microarray probes was synthetized by one cycle of RNA amplification in which molecules were labeled in an Affymetrix microarray station (Affymetrix, CA). Labeled microarray probes were hybridized on a Mouse Clariom S (mm10) microarray (Thermo Fisher Scientific). The CEL files of microarray data obtained from the Affymetrix platform were normalized using the RMA method in Affymetrix Expression Console software (Affymetrix, CA) (34). As described by Lyons et al. (35), gene modules of specific immune cell expression profiles were constructed for the purpose of immune-cell-specific profiling of cancer. With these specific immune modules, we conducted gene-set enrichment analyses using the online java module of GSEA software version 3.0 (36). A network-based analysis of gene enrichment was performed with Cytoscape software version 3.6.0 (31). Differentially expressed genes were identified with a rank products analysis. A functional analysis of genes that were upregulated in the vaccinated group was performed using the Gene Ontology biological process database and the DAVID application from the NIH website (37).

### qRT-PCR

Total RNA was isolated using TRIzol Reagent (Life Technologies) and reverse transcription was performed using MultiScribe Reverse Transcriptase (Applied Biosystems) according to the manufacturer's instructions. Quantitative PCR (qPCR) was performed in duplicate using SYBR Green PCR Master Mix (Applied Biosystems) on an Agilent Technologies Stratagene MX3005p apparatus. The expression levels of genes were normalized to that of glyceraldehyde-3-phosphate dehydrogenase (GAPDH). The sequences of the primers used are: for CXCL9: Fw: CCATGAAGTCCGCTGTTCTT, Rv: TGAGGGATTTGTAGTGGATCG, for CXCL10: Fw: ATCAGCACCATGAACCCAAG, Rv: TTCCCTATGGCCCTCATTCT, for CXCL13: Fw: ATGAGGCTCAGCACAGCA, Rv: ATGGGCTTCCAGAATACCG, for GAPDH: Fw: GAGAGGCCCTATCCCAACTC, Rv: TCAAGAGAGTAGGGAGGGCT.

### Quantification and Statistical Analyses

All values are expressed as mean ± s.e. Differences among groups were assessed, as appropriate, using either an unpaired two-tailed Student's *t*-test or a one-way/two-way analysis of variance (ANOVA) in PRISM GraphPad software or Microsoft Office Excel software. ^*^P < 0.05, ^**^P < 0.01, ^***^P < 0.001, ^****^P < 0.0001.

## Results

### Poorly Differentiated Murine 4T1 Breast Tumors Display an IPSCs-Like Expression and Stemness Signature

**Figure 1:**
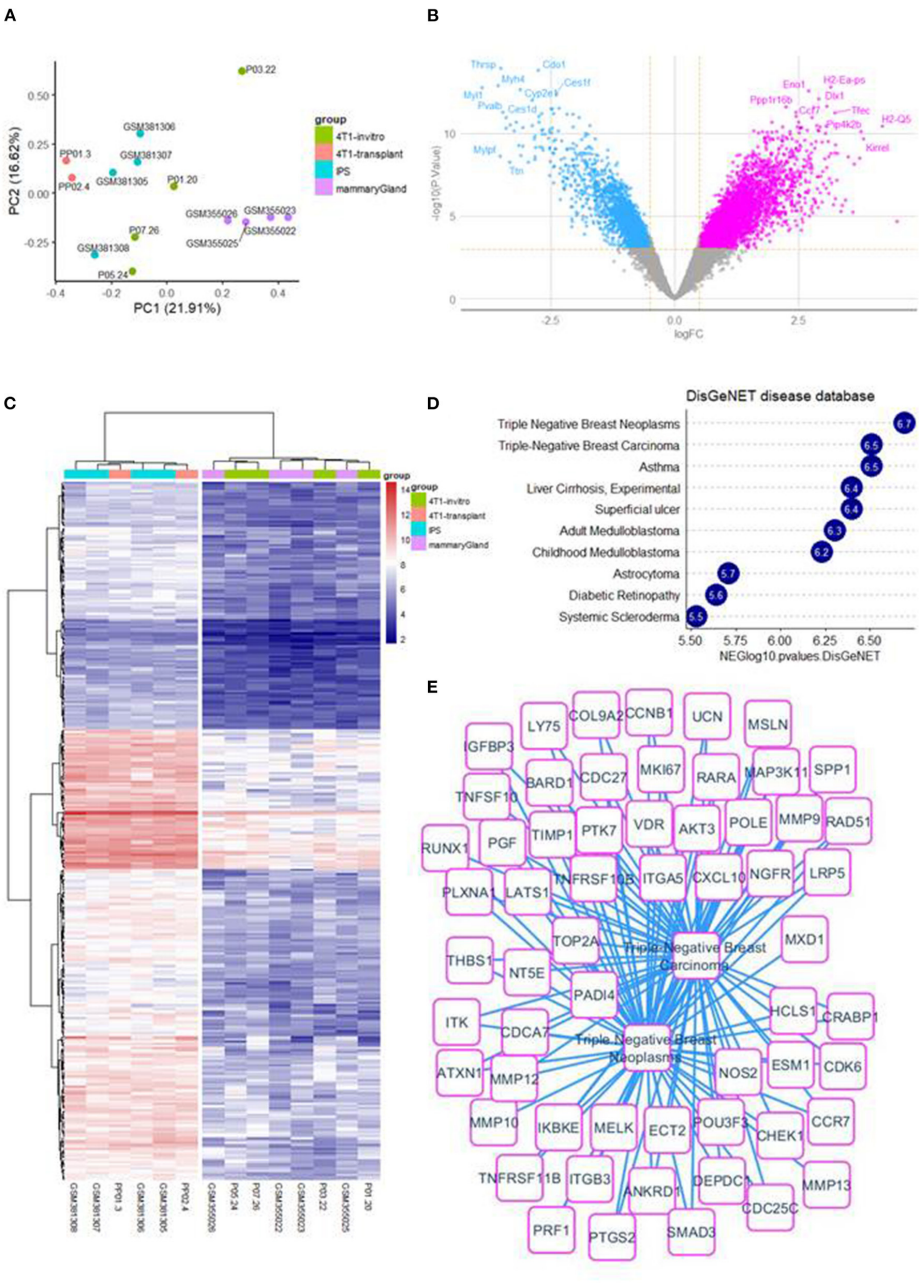
4T1-transplant samples involved a TNBC up regulated expression profile: **(A)** whole transcriptome principal component analysis stratify on experimental groups. **(B)** Volcanoplot of LIMMA analysis comparing 4T1-transplant and 4T1 *in vitro*. **(C)** Expression heatmap on up regulated genes between 4T1-transplant and 4T1 *in vitro* condition. **(D)** Barplot of functional enrichment performed on DisGeNET with up regulated genes in 4T1-transplant condition. **(E)** Functional enrichment network involving triple negative breast cancer expression profile for 4T1-transplant up regulated genes.

Cross batch normalization, transcriptome integration of 4T1 *in vitro* and 4T1-transplant samples with samples of mouse iPSCs and mouse micro dissected mammary gland (Figure 1A) have allowed to perform a differential expression analysis that was performed with LIMMA algorithm between 4T1-transplant and 4T1 *in vitro* (Figure 1B). This supervised analysis allowed to identify 325 up regulated genes in 4T1-transplant condition (Log_2_ Fold Change >2 and Adjusted *p* < 0.01, Supplementary Table 2). Unsupervised clustering performed with up regulated genes in 4T1-transplant allow to aggregate miPSCs samples with 4T1 transplant samples in one cluster and 4T1 *in vitro* samples with micro-dissected mammary gland samples in another cluster (Euclidean distance and ward.D2 method, Figure 1C). Functional enrichment analysis performed with these 325 up regulated genes in 4T1-transplant on DisGeNET database allowed to identify a main enrichment in triple negative breast cancer disease (Figure 1D). Triple negative breast cancer enriched signature in 4T1-transplant condition allowed to build a large molecular related network (Figure 1E). In addition, enrichment on GO-BP database allowed enrichment of terms in relation with development and morphogenesis and especially genes implicated in epithelial and tube development which are components important for mammary gland pathophysiology (Supplementary Figures 1A,B). Functional enrichment performed on MSigDb version 7.3 allowed also to identify an enriched molecular network implicating relations with microenvironment such as: focal adhesion kinases, integrin and extracellular matrix organization with several collagens and metalloproteinases (Supplementary Figures 1C,D) as well in cell mobility and migration (Supplementary Figures 2A,B). All these functional enrichments are in agreement with an invasive process and with a mesenchymal characteristic of this TNBC mouse model after injection in mice.

We then used FACS analysis to quantify the expression of the breast cancer stem cell markers CD44 and CD24 (38) in 4T1 cells recovered *in vitro* and *in vivo* 12 and 28 days after injection into mammary fat pads. A population of CD44^+/high^/CD24^−/*low*^ cells was identified in 4T1 cells *in vivo*, with a frequency up to 32% (Supplementary Figure 3). Since it is well-known (38) that induction of an EMT in transformed mammary epithelial cells yields cells with CD44^high^/CD24^low^ antigenic phenotype, we wished to confirm the presence of EMT markers by whole transcriptome unsupervised principal component analysis.

### Valproic Acid Modify the Transcriptomic Landscape of 4T1 Cells

**Figure 2:**
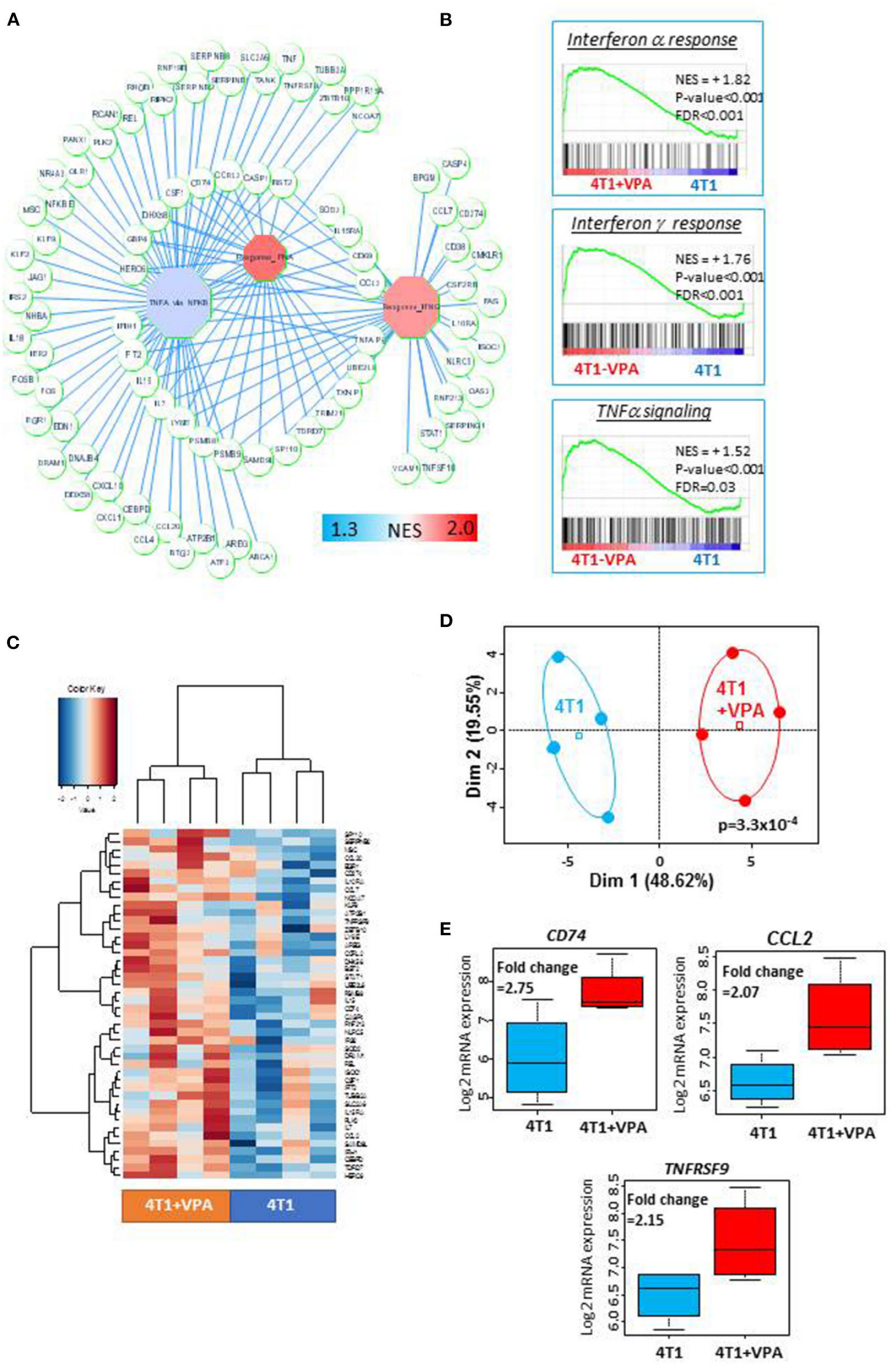
Treatment with valproic acid (VPA) induced an upregulation of the immune response in 4T1 cells *in vitro*: Transcriptome analysis was performed on 4T1 cells treated with or without 0.5 mM of valproic acid for 10 days. Microarray probes were synthetized, labeled (Affymetrix microarray station, Affymetrix, CA) and hybridized on a Mouse Clariom S microarray (Thermo Fisher Scientific). The CEL files of microarray data obtained from the Affymetrix platform were normalized using the RMA method in Affymetrix Expression Console software (Affymetrix, CA). **(A)** A representation of the immune gene network upregulated by *in vitro* VPA treatment of 4T1 cells. Octagons represent enriched immune modules; circles are genes, which are connected to their respective enriched modules by blue edges (NES, normalized enrichment score). **(B)** Immune gene sets that were significantly enriched in VPA-treated 4T1 cells compared to untreated 4T1 cells (NES, normalized enrichment score, *p*-value and FDR were obtained by a hypergeometric test performed using the MSigDB 6.1 Hallmark database). **(C)** Expression heatmap depicting the immune genes that were significantly overexpressed in VPA-treated 4T1 cells (SAM algorithm, unsupervised classification on Euclidean distances with Ward method). **(D)** Unsupervised principal component analysis performed on upregulated immune genes in VPA-treated 4T1 cells. **(E**) Boxplot of three immune genes that were found to be significantly overexpressed as a result of VPA treatment (fold-change >2).

Before testing the *in vivo* immune-modulatory effect of VPA in combination with iPSC-based vaccine, we asked whether VPA could modulate on its own, the immune-related gene expression in 4T1 cells. To this purpose, we performed a transcriptome analysis on 4T1 cells treated *in vitro* with 0.5 mM of VPA for 10 days, and compared this to the transcriptome of untreated cells. These analyses identified 117 immune-related genes implicated in TNF alpha and/or IFN-alpha and IFN-gamma signaling (Figure 2A). These results were confirmed by a gene-set enrichment analysis that revealed significant enrichment in these three immune gene sets (Figure 2B). In addition, using the SAM algorithm we were able to identify 44 immune-related genes that demonstrated expression differences between the VPA-treated samples and their control counterparts (Figure 2C, Supplementary Table 3). These were validated by principal component analysis (Figure 2D, *p* = 3.3 × 10^−4^). Among these 44 immune genes, *CD74, CCL2*, and *TNFRSF9* had a fold-change expression of >2 (Figure 2E, Supplementary Table 3). In addition, we discovered that VPA could increase the MHC I expression level in a dose-dependent manner (Supplementary Figure 4A) with 2 mM of VPA exposure inducing a 2.1-fold increase expression of MHC I (relative mean of fluorescence intensity measured by FACS). More importantly, VPA also induced a 2.7-fold increase expression of MHC I in 4T1-derived MammoSpheres (MSs) (Supplementary Figure 4B) which harbored a high proportion of CSC-like cells expressing aldehyde dehydrogenase 1 (ALDH1) in permissive culture conditions, in the presence of high doses of TGFβ and TNFα (Supplementary Figures 5A–D).

The finding of MHC I expression in “stemness” conditions, led to the hypothesis that VPA treatment could promote directly immune response-associated gene expression, which would improve immune-recognition of CSC-like 4T1 cells by T-cells.

To test this hypothesis, we wished to eliminate the possibility that VPA alone had an effect on tumor growth. We therefore performed tumorigenicity experiments in which we have evaluated the effects of VPA alone on 4T1 tumor size or on metastatic spread to the lungs. In the experiments using VPA alone, there was no significant effect on tumor reduction (*p* = 0.73; Supplementary Figures 6A,B), nor on the occurrence of metastatic spread (*p* = 0.54; Supplementary Figure 6C) and no effect on CD4^+^ or CD8^+^ T-cells, Tregs or MSCS (Supplementary Figure 6D). These data suggested that VPA alone in this dose was insufficient for inducing an immune response against the 4T1 model of breast cancer.

These results prompted us to investigate a combinatory immune strategy using an iPS cell-based cancer vaccine along with the VPA.

### Anti-tumor Effects of Murine iPS Cell-Based Vaccines Combined With VPA in an Autologous or Allogeneic Context

To confirm the hypothesis of the occurrence of the immune response along with the anti-tumor effect using the combined strategy, we immunized BALB/c immunocompetent mice with the vaccine combination approach, involving 2-weekly 2 × 10^6^ allogenic C57BL/6 derived-miPSCs pre-treated by VPA followed by transplantation of 4T1 cells into the fad pad (Supplementary Figure 7). After implantation of the tumor, mice were treated orally by VPA (4 mg/mL) during 15 days. Anti-tumor efficacy of this immune prevention strategy was compared with a preventive therapy using C57BL/6-derived miPSCs without VPA treatment and with VPA treatment alone without vaccination.

**Figure 3:**
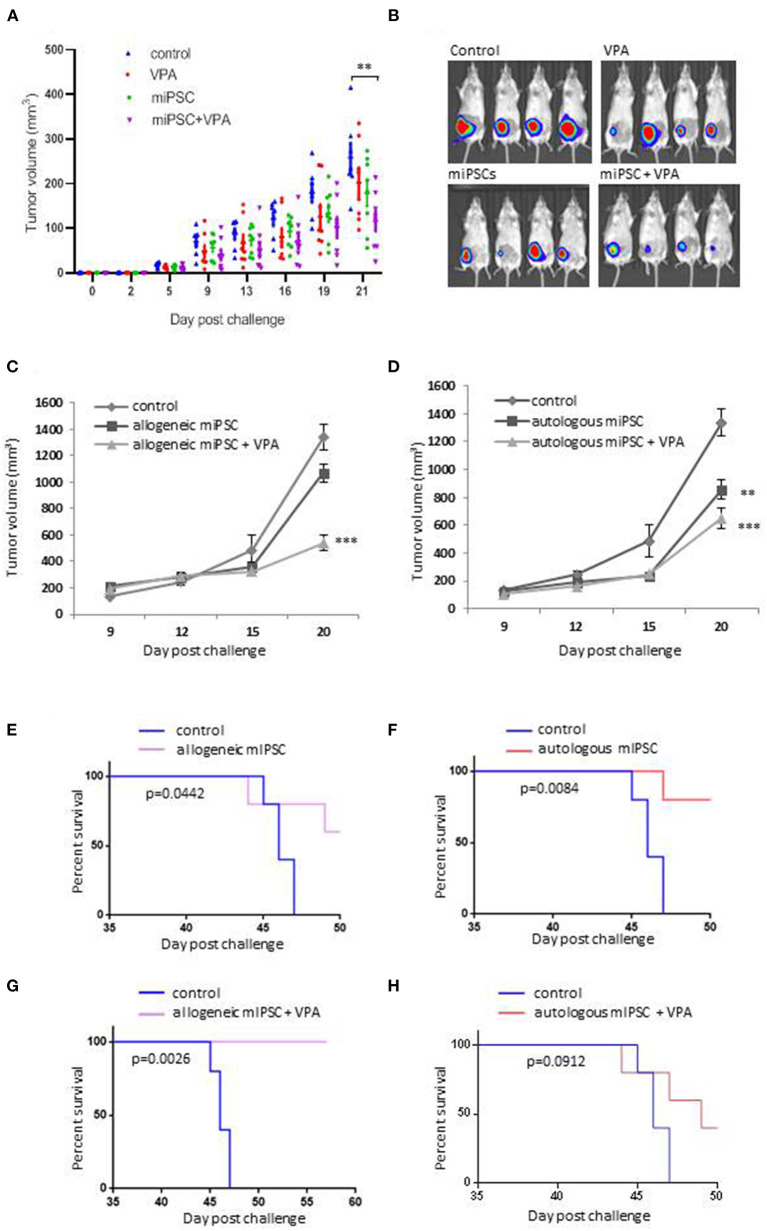
Evaluation of the anti-tumor effects of murine iPSCs derived from BALB/c or C57BL/6 mice. **(A)** Evaluation of the anti-tumor effects of C57BL/6- derived miPSCs murine with or without VPA. Eight to 10 weeks old female BALB/c mice, were divided into 4 groups: group control (PBS; *n* = 7), mice treated with VPA (*n* = 8) or miPSCs alone (*n* = 8), and mice treated with miPSCs + VPA (*n* = 8). Treatment consisted of two sub-cutaneous injections (one-week interval between injections) of 2 × 10^6^. One week following the second injection, all mice were inoculated with 5 × 10^4^ 4T1Luc cells into mammary fat-pad following by 21 days of VPA treatment, orally administered at dose of 4 mg/mL. **(B)** Representative bioluminescence imaging of tumors from mice treated with miPSCs, VPA and miPCs+VPA compared to untreated mice. **(C)** Tumor growth in 8–10 weeks old, BALB/c mice that were immunized with murine C57BL/6-derived iPSCs; with (*n* = 5) or without VPA (*n* = 5), compared to control group (*n* = 5) by using the same protocol as previously. **(D)** Tumor growth in 8–10 weeks old, BALB/c mice that were immunized with murine BALB/c-derived iPSCs; with (*n* = 5) or without VPA (*n* = 5), compared to control group (*n* = 5) by using the same protocol as previously. **(E)** Effect of allogeneic C57BL/6-derived miPSC treatment on the survival of mice BALB/c challenged with 5 × 10^4^ 4T1 cells (*n*=5 per group). **(F)** Effect of autologous BALB/c-derived miPSC treatment on the survival of BALB/c mice challenged with 4T1 cells 5 × 10^4^ 4T1 cells (*n*=5 per group). **(G)** Effect of allogeneic C57BL/6-derived miPSC +VPA treatment on the survival of mice BALB/c challenged with 5 × 10^4^ 4T1 cells (*n* = 5 per group). **(H)** Effect of autologous BALB/c-derived miPSC treatment on the survival of BALB/c mice challenged with 4T1 cells 5 × 10^4^ 4T1 cells (*n* = 5 per group). ** *p* < 0.01, *** *p* < 0.001.

Overall these experiments were conducted using 31 BALB/c mice divided into four groups including PBS group (*n* = 7), VPA alone group (*n* = 8), C57BL/6 derived-miPSCs group (*n* = 8) and the group of mice treated with C57BL/6 derived-miPSCs and VPA (*n* = 8). All mice were challenged with 5 × 10^4^ 4T1-GFP-Luc cells. At day + 21 after tumor implantation, the group of mice treated with VPA alone and miPSC-based vaccine alone showed 21.7 and 18% reduction, respectively, in their tumor volume which was not significant as compared to those observed in control mice, On the other hand, in mice receiving miPSC with VPA, the tumor volume was significantly smaller with a reduction of 47.6% compared to control mice (104 ± 5.7 vs. 258 ± 12 mm^3^, *p* < 0.001; Figures 3A,B).

Vaccination experiments were then investigated (i) to explore the effectiveness of the combinatory miPSCs + VPA approach as compared to the regimen using miPSCs alone on tumor size, survival rate and metastases, and (ii) to compare allogeneic C57BL/6-derived miPSCs based vaccine with autologous BALB/c-derived miPSCs.

To this purpose we have used miPSC from BALB/c fibroblasts (Supplementary Figure 8A) which exhibited classical pluripotency characteristics (Supplementary Figures 8B,C) and generated teratomas *in vivo* (Supplementary Figure 8D). Naïve immunocompetent BALB/c mice were pre-immunized every 2 weeks with 2 × 10^6^ allogeneic or autologous miPSCs with or without VPA treatment followed by tumor challenge. Negative control group was injected with PBS without VPA treatment.

As shown in Figures 3C,D, the combinatory treatment with miPSCs and VPA allowed a better reduction in the tumor burdens, as compared to control as well as mice vaccinated without VPA treatment. We observed a higher tumor reduction in mice treated with allogeneic miPSC + VPA (61% of reduction; *p* < 0.0001) as compared to mice treated with autologous miPSC + VPA (48% of reduction; *p* = 0.0018).

Experiments evaluating the long-term survival (>50 days) showed that vaccination with miPSC conferred a significant improvement in survival rate over the control group (Figures 3E,F). The most significant survival benefit was observed when VPA was combined with the allogenic vaccine (*p* = 0.0026, 100% of survival) (Figure 3G). In contrast survival rate was less pronounced with the autologous vaccine combined with VPA (*p* = 0.0912; Figure 3H).

We confirmed the efficacy of the combination regimen of allogenic miPSC and VPA in BALB/c mice that were treated using the same protocol as previously by monitoring the tumor volume until day +20, in vaccinated mice (C57BL/6-derived miPSCs + VPA; *n* = 8) and unvaccinated mice (*n* = 7) (Supplementary Figure 9). After 3 days, all mice from both groups presented tumors of similar size. Tumors from control mice grew dramatically over the remainder of the experiment. Instead, in mice treated with allogeneic C57BL/6-derived miPSC+VPA, tumors grew much more slowly, and indeed, even shrank in 4 of the 8 treated mice. In one mouse, the tumor completely disappeared, while the other three mice demonstrated a partial regression in tumor size, with final volumes of <35 mm^3^ (Supplementary Figure 9).

### Iterative MiPS-Cell Based Vaccines Promote an Efficient Memory Immune Response to Prevent Tumor Growth and Metastatic Spread

**Figure 4:**
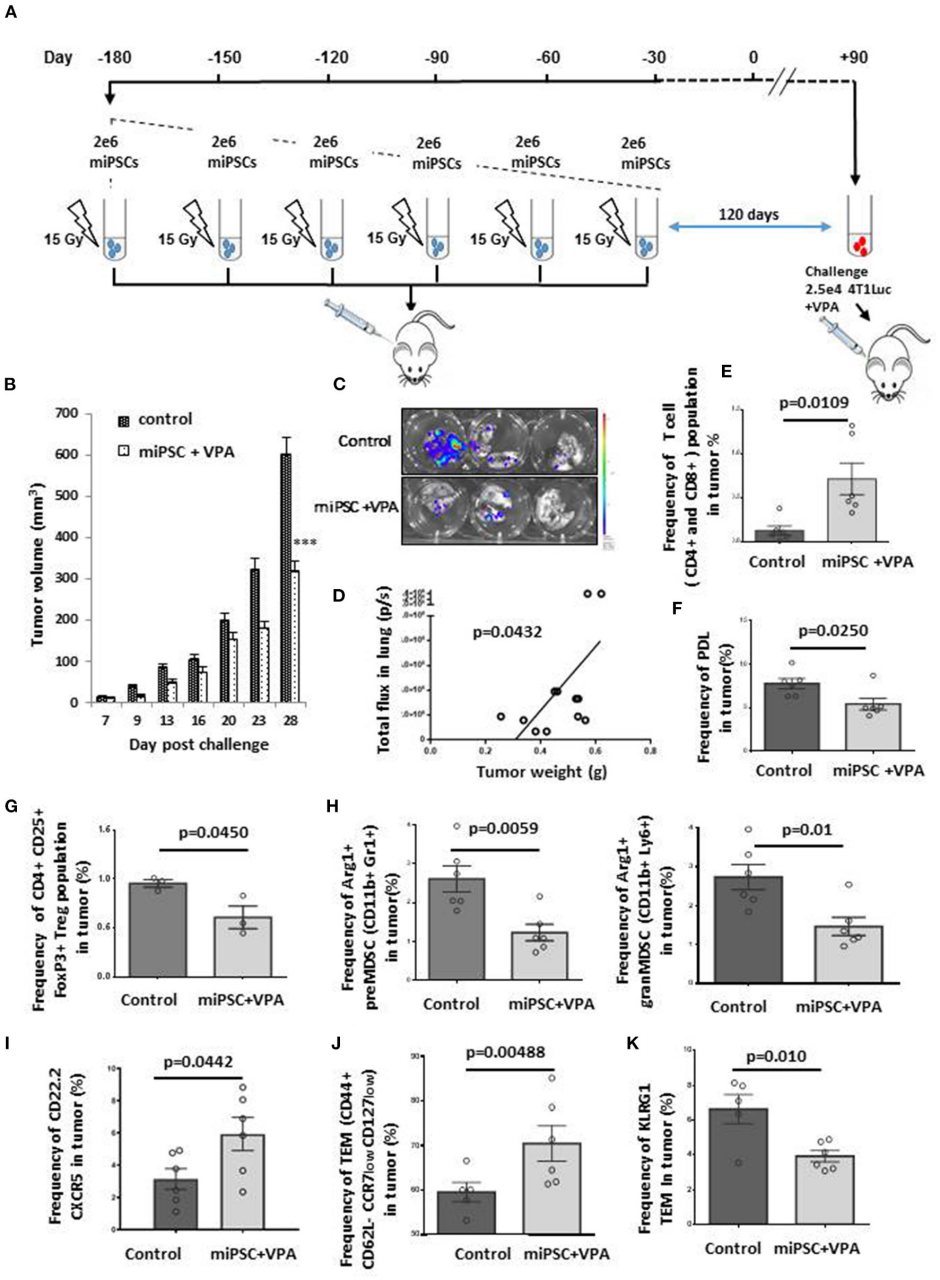
Effective memory immune response following vaccination with autologous miPSCs. Mice were treated (*n* = 6) with sub-cutaneous injections of 2 × 10^6^ irradiated BALB/c-derived miPSCs during 6 months, with a total of 6 injections, once every 30 days. One hundred twenty days after the last vaccine inoculation, mice were challenged with 2.5 × 10^4^ 4T1Luc cells and vaccinated mice received 4 mg/mL VPA by oral route starting from the day of tumor injection until the sacrifice. Control mice (*n* = 7) received only PBS. **(A)** Experimental protocol to evaluate *in vivo* immune memory generated by vaccination: BALB/c mice were injected subcutaneously six times with 2 × 10^6^ miPSCs (15 Gy irradiated) in the right flank. **(B)** At day 28 post-challenge, breast tumors were significantly smaller in mice that had undergone the six-month vaccination protocol compared to unvaccinated mice. **(C)** Bioluminescence images of lungs isolated from miPSC-vaccinated and control mice 28 days after tumor challenge. **(D)** A significant correlation was found between tumor burden and metastatic spread in the lungs of vaccinated mice at day 28 post-challenge. **(E)** The frequency of CD4^+^CD8^+^ cells in tumors of treated and untreated mice, as measured by flow cytometry. **(F)** The frequency of PDL1^+^ cells in tumors of miPSC-vaccinated mice compared to controls, as measured by flow cytometry. **(G)** The frequency of Treg cells in tumors of miPSC-vaccinated mice compared to controls, as measured by flow cytometry. **(H)** The frequency of Arg1^+^ preMDSCs and granMDSCs in tumors of miPSC-vaccinated mice compared to controls, as measured by flow cytometry. **(I)** The frequency of CXCR5^+^ CD22.2^+^ LB cells in tumors of miPSC-vaccinated mice compared to controls, as measured by flow cytometry. **(J)** The frequency of T-effector memory cells in tumors of miPSC-vaccinated mice compared to controls, as measured by flow cytometry. **(K)** The frequency of KLRG1^+^ T-effector memory cells in tumors of miPSC-vaccinated mice compared to controls, as measured by flow cytometry, *** *p* < 0.001.

We then asked whether miPS-cell based vaccine could be used in healthy mice treated with iterative injections aiming to induce a durable long-term T-cell memory response. For this purpose, we have chosen to use autologous miPSC to avoid allo-immune response. We inoculated mice with 2 × 10^6^ irradiated BALB/c-derived miPSCs during 6 months, with a total of 6 injections, once every 30 days. Thirty days (Supplementary Figure 10A) or 120 days (Figure 4A) after the final vaccine inoculation, mice were challenged with 2.5 × 10^4^ 4T1Luc cells. All vaccinated mice received 4 mg/mL VPA by oral route starting from the day of tumor injection, and tumor growth was monitored for 26 or 28 days after challenge. All mice primed by miPSCs cells were healthy without any side effects after 6 months of vaccination, which suggest that the protocol proposed was a safe preventive approach.

Indeed, tumor volume was reduced by 64% in mice challenged with 4T1 30 days after the final vaccine dose, compared to unvaccinated mice (695 ± 102 vs. 1,968 ± 96 mm^3^, *p* = 0.005; Supplementary Figure 10B). When we increased the time elapsed between the final vaccine dose and tumor implantation to 120 days, we observed again a significant response (46% reduction: 320 ± 23 vs. 600 ± 40 mm^3^ for controls, *p* < 0.001; Figure 4B).

We performed an in-depth investigation of the tumor dissemination status in mice for which 120 days elapsed between their final vaccine dose and tumor challenge. The analysis of metastatic dissemination at + 28 days, revealed a major reduction in the lung metastases in this group compared to controls (Figure 4C), with a significant correlation between tumor burden and metastatic spread (Figure 4D).

In both cases, repeated doses of miPS-whole cell vaccines mediated a tumor effective immune response that resulted in a significant inhibition of tumor growth and metastatic spread compared to the control group.

We then analyzed the tumors with regard to their tumor infiltrating lymphocytes (TILs) contents and tumor immune micro-environment (TIME) landscape. Treatment with miPSCs combined with VPA was correlated with a significant increase in the frequency of CD4^+^ or CD8^+^ T cells in the tumors (Figure 4E) and in the spleens (Supplementary Figures 11A,B). Furthermore, we observed a significant decrease in the frequency of PD1^+^ CD4^+^ cells in the spleen (Supplementary Figure 11C), as well as a decrease in PDL1 expression in tumor cells (Figure 4F), suggesting strongly that the vaccination protocol had overcome the T-lymphocyte anergy.

The analysis of the cellular TME components showed that the combined miPSCs and VPA administration was able to convert the immune- repressive TME into an active one by a significant decrease of the CD4+ CD25+FoxP3+ Tregs frequency (Figure 4G) as well as the decrease of Arg1^+^ CD11b+ Gr1+ pre-MDSCs and Arg1^+^ CD11b+ Ly6+ gram MDSCs frequency (Figure 4H) which are the main immunosuppressive actors of the TME.

Repeated doses of autologous miPSC were found to mediate additional long-term effects on CD22.2^+^ B-lymphocytes. Specifically, we observed a significant increase in CXCR5^+^ B cells in vaccinated mice as compared to controls (Figure 4I), suggesting that B-lymphocytes had migrated to tumor sites as a result of the combined therapy. Level of T-Effector Memory Cells (TEMs) was also evaluated and showed an increase of CD44+ CD62L- CCR7low CD127low TEMs frequency in the tumors (Figure 4J). The strong decrease of KLRG1 expression on TEMs into the tumors (Figure 4K) and the decrease of PD1 expression by TEMs (CD44+CD62L-CCR7 low CD127 low) from spleens (Supplementary Figure 11D) suggested that the immune memory had reverted to an active state, decreasing both senescence and anergy.

These results provide strong evidence that a sequential miPS- whole cell based vaccination led to the establishment of long-term immune memory without any side effects with the activation of an effective anti-tumor immunity against metastatic spread. A strong benefit was also seen in survival data, as our vaccination protocol dramatically improved the median long-term survival of treated mice as compared to controls (54 days in control group vs. not reached in vaccinated group) (Supplementary Figure 10C).

Finally, we wished to compare the efficacy of the vaccination protocol against the tumor challenge by 2.5 × 10^4^ MammoSpheres-derived 4T1 cells (Supplementary Figure 12A). As we found with our previous results, immunization with miPSCs led to a significant (*p* < 0.0001) and highly effective immune response to MS-derived 4T1 cancer cells. The total tumor burden in treated mice was reduced by 83% compared to controls, with a massive reduction in tumor sizes (Supplementary Figures 12B,C). The long-term immune memory generated by our protocol also significantly protected the mice from developing lung metastases (Supplementary Figures 12D,E).

**Figure 5:**
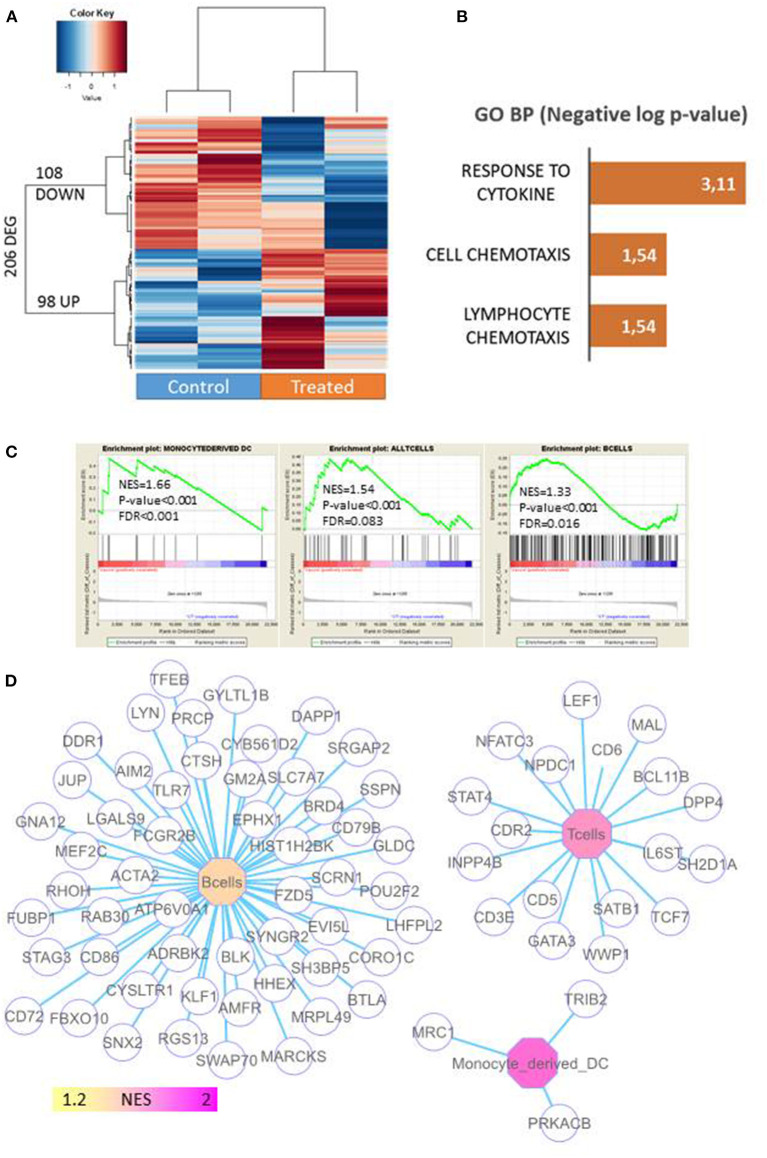
Profiling of immune-associated genes in 4T1 tumors in mice primed with miPSCs. Transcriptome analysis was performed on tumors from untreated mice and from mice primed with 6 doses of miPSCs and VPA in order to find genes that were differentially expressed between the two conditions. Total RNA was extracted from 4T1 tumors in treated and control mice, following the instructions of the manufacturer (TRIzol, Life Technologies). Microarray probes was synthetized by one cycle of RNA amplification in which molecules were labeled in an Affymetrix microarray station (Affymetrix, CA). Labeled microarray probes were hybridized on a Mouse Clariom S (mm10) microarray (Thermo Fisher Scientific). **(A)** Differentially expressed genes (DEGs) between control and vaccinated mice xeno-transplanted with 4T1 cancer cells; expression heatmap was produced with an unsupervised classification algorithm (Euclidean distances, Ward method). **(B)** Barplot of functional enrichment in Gene Ontology Biological Processes of genes that were overexpressed in vaccinated mice. **(C)** Immune profiling carried out by gene-set enrichment analysis on the transcriptome of 4T1-transplanted mice (NES: normalized enriched score). **(D)** Genes associated with the immune network that were enriched in 4T1 tumors from miPSC+VPA-treated mice.

To investigate the underlying mechanisms of the long-term immune protection that resulted from the vaccination protocol, we performed a transcriptome analysis of tumors from untreated mice and from mice primed with 6 doses of miPSCs and VPA in order to find genes that were differentially expressed between the two conditions. As can be seen in Supplementary Table 4 and Figure 5A, 206 genes were found to be differentially expressed in a highly significant manner.

Of the genes that were upregulated in treated mice, 98 were implicated in the immune response to cytokines and lymphocyte chemotaxis (Figure 5B). When we analyzed genes linked to the immune response, we detected a significant enrichment in monocyte-derived dendritic cells [Normalized Enriched Score (NES) = 1.66, *p* < 0.001], T cells (NES = 1.54, *p* < 0.001), and B cells (NES = 1.33, *p* < 0.001; Figure 5C). Likewise, a gene network-based analysis supported the major contribution of B-cells and T-cells by highlighting genes associated with these respective infiltrations (Figure 5D). Interestingly, a significant upregulation (8.8-fold; *p* = 5.1787803E-5) of chemokine (C-X-C motif) ligand 13 was detected in tumors that persisted after vaccination (Supplementary Table 4). This result was confirmed by qRT PCR, which also revealed a strong upregulation of CXCL13, CXCL9, and CXCL10 in the tumors of vaccinated mice (Supplementary Figure 13).

## Discussion

In this study, we evaluated for the first time the effects of a combinatory vaccination strategy using autologous and allogeneic iPS-whole cell-based vaccine along with a histone deacetylase inhibitor (HDACi) in cancer development in an aggressive murine breast carcinoma model with metastatic potential. Cancer-stem like cells escape to immune system by different mechanisms in particularly by deregulation of signaling pathways and the silencing of the MHC I expression by several epigenetic perturbations. In order to promote the immune-recognition of cancer cells, our vaccination strategy used the addition of VPA, an HDACi which removes acetyl marks from tagged histones to increase global histone acetylation. Interestingly, HDACi might also work to re-activate gene expression by altering the global nuclear architecture. Thus, increase in histone acetylation can result in a relaxed chromatin configuration, enabling access to transcriptional activators to restore gene transcription. Epigenetic drugs targeting these enzymes can restore, and in some cases induce overexpression of genes that have been epigenetically silenced in both immune and cancer cells (39) including MHC1 molecules (40).

The concept of using iPSCs as a source of tumor-associated antigens (TAAs) (41, 42) with the ultimate goal of eliciting an anti-tumor immune response was previously reported either on the preventive effects of embryonic stem cells on transplantation of cancer cell lines (16–18) or using the autologous anti-tumor vaccines using iPSCs, with TRL9 as adjuvant, in a prophylactic setting in a non-metastatic syngeneic murine cancer cell lines (14, 15). However, none of these existing reports evaluated the anti-metastatic potential of iPS-whole cell vaccines, nor did they determine whether such vaccines had the ability to target aggressive tumors enriched with CSCs-like cells and to modify their microenvironment.

We show in this work for the first time that a combined iPS-cell-based cancer vaccine and HDACi (here valproic acid) strategy was highly efficient to inhibit the growth of an aggressive, poorly differentiated triple-negative breast cancer (TNBC) cell line. This model closely mimics the metastatic human basal-like breast cancer responsible for rapid and lethal metastatic spread *via* a hematogenous route mainly to the lungs (43, 44).

We also uncovered in this work the efficacy of this iPS-cell-based cancer vaccine for its broad mechanism of action by immune modulation effect and its multiple immune stimulatory functions able to modify the tumor microenvironment.

Because the antigen processing ability of CSCs is epigenetically down-regulated, they express low levels of MHC-I molecules, leading to their altered detection by the host immune system (45). This effect is further enhanced by the fact that the surrounding tumor microenvironment is highly immunosuppressive (45). The combination of these two factors is remarkably effective in enabling CSCs to escape the surveillance of an efficient immune system.

We have also explored in this work, the potential of HDACi to increase the efficacy of the miPS-cell-based cancer vaccine. HDACi are known to have multiple biologic effects consequent to alteration in patterns of acetylation of histones, implicating proteins involved in the regulation of gene expression, pathways of apoptosis, cell cycle progression, mitotic division, cell migration, and angiogenesis (46). HDACi were also shown to have potent immunomodulatory activities. There are robust data supporting the use of epigenetic drugs such as HDACi on their ability to modulate immune-cancer cell interactions leading to the reversal of crucial events of immune evasion. Indeed, HDACi were shown to increase the expression of TAAs and specially Cancer Testis Antigens (CTAs) (47) and to increase the expression of perforin in T cells (48). They also increase the antigenic recognition by CTLs (49) by mediating recognition of cancer cells by CTLs (50). It has also been demonstrated that HDACi down regulate MDSC expansion and function, reduce the expression of arginine-1, which are known to impair T cell proliferation and cytokine production (51) and to enhance T-cell chemokine expression (52). HDACi have also been proven to modulate innate host immune cells by increasing the expression of the activating receptor NKG2D on the surface of NK cells (53). It has been previously shown that they enhance NK cell-mediated tumor cell targeting by upregulating the stress-inducing ligands MICA, MICB, and ULBP1-3 in tumor cells permitting a more efficient killing of tumor cells (50). In addition, they have been reported to increase the expression of death-inducing receptors FAS and TRAIL-R2 on cancer cells enhancing the death of cancer cells by NK cells (54).

We observed in our experimental context, that VPA had various mechanisms of action, allowing to improve cancer vaccine efficacy by transforming the TME and to overcome the immunological tolerance of the cancer stemness phenotype by drastically altering the immune system within the TME. Several lines of finding support this assumption. Firstly VPA had the potential to significantly increase the expression of MHC1 in 4T1 cells and in CSC/MS-derived 4T1 cells. It has also increased the expression of MHC2 (CD74), chemokine CCL2, and TNFRSF9, a member of the TNF-receptor superfamily that is known to contribute to the clonal expansion, survival, and development of T cells and to regulate CD28 co-stimulation to promote Th1 cell responses. CCL2 is a chemokine shown to be a potent chemo attractant for several types of immune cells, including NK cells, memory T cells, and dendritic cells within the tumors (55). We also determined according to other observations (40), that VPA promoted the production *in vivo* of multiple chemokines, such as CXCL9, 10, and 13, which enabled significant modifications in the immunosuppressive microenvironment of tumor cells and facilitated the local recruitment of T and B cells within the tumor. VPA was also found to attenuate the immunosuppressive proportion of myeloid-derived suppressor cells (MDSC). This last mode of action is in concordance with previous studies showing that HDACi treatments efficiently decrease Gr-1+ MDSC accumulation in the spleen and tumor bed in BALB/C mice with 4T1 mammary tumors by inducing MDSC apoptosis through mitochondrial Reactive Oxygen Species (ROS) signaling pathway (56).

One important discovery reported here is related to the possibility of generating anti-cancer immunity not just toward “bulk” cancer cells but also toward primitive de-differentiated CSC-like MammoSphere cells. Indeed, in several cancers, gene expression programs have been identified to be similar to those of embryonic pluripotent stem cells, namely, a “stemness” profile which is associated to oncogenic de-differentiation in epithelial cancer progression by a gradual loss of a differentiated phenotype and acquisition of progenitor and stem cell-like features (7, 8, 57).

It is well-established that CSCs are the cause (i) of resistance to “classical” therapies (58–60), (ii) of relapses in several types of aggressive cancers (61–64) and (iii) of metastatic dissemination (65). Tumor cells undergoing EMT are enriched in CSCs with a capacity for early migration and long-term persistence in a dormant stage for long periods of time. Such cancers correlate with aggressive, poorly differentiated tumor histology, invasive tumors, and very adverse outcomes (7, 8, 66).

Prior to our study, there had been no evidence presented as to whether immunotherapy vaccine strategies that use iPSCs as the source of TAAs have the ability to specifically target CSCs and/or the CSC niche in TNBC. The 4T1 breast cancer model is well suited for addressing this question as it is known to hijack some of the normal stem cell pathways to increase cellular plasticity and stemness (44). Our combinatory regimen iPSCs + VPA as adjunct had a substantially enhanced anti-tumor effect compared to the vaccine-only treatment or to the use VPA alone, and caused a significant reduction in lung metastases. These results indicate that iPSCs + VPA have significantly modified the immunosuppressive microenvironment within the primary tumor and reduced the number of cancer cells with a stemness/CSC phenotype that were able to migrate to the secondary organs.

In this work, we evaluated two sources of fibroblast-derived iPSCs generated from the BALB/c and C57BL/6 strains of mice, respectively; this enabled us to compare anti-tumor immunity generated in BALB/c mice under autologous vs. C57BL/6 allogeneic conditions. Regardless of the strain of origin, vaccinated mice had significantly smaller tumors compared to unvaccinated controls, and the inclusion of VPA treatment increased the anti-tumoral response considerably. We also found a significant improvement in the survival rate due to vaccination, and this was more pronounced in mice that had been primed with allogeneic material combined with VPA. Allogeneic iPSCs trigger a stronger cellular and humoral alloimmune response due to allo-immunity stimulated by MHC mismatches.

The use of allogenic iPSCs represents a considerable advantage in the development of a scalable cancer stem cell-based vaccine for all solid tumors with stemness feature. Allogeneic iPSCs allow the development of “off-the-shelf” whole cell-based vaccine for curative approaches combined with other therapies. In our experiment design, this allogeneic iPSC-based therapy could be used as an adjuvant approach, in order to prevent the short and long-term risk of relapse and metastasis. Our allogeneic iPSCs-whole cell-based vaccine with VPA could be used at distance of any chemotherapy, in minimal residual disease or in patients with apparent remission to eradicate residual CSCs in multiple cancer sharing stemness feature.

The length of time needed to produce bona fide iPSCs (several months, corresponding to several passages in culture and quality controls to ensure their purity and safety) would render autologous iPSCs highly impractical for curative treatment. However, in patients harboring germline mutations with a high risk of cancer, autologous-derived iPSCs would likely be useful in prophylactic settings. We confirmed the safety of iterative repeated doses of autologous miPSC injection in healthy immune-competent mice, which allowed the generation of a pool of effective memory T cells and B cells against 4T1 breast carcinoma development. Indeed mice that were challenged with 4T1 cells 30 or 120 days after the end of a six-dose vaccination series were able to reject 4T1 cells. These anti-tumoral responses was correlated with a significant increase in the frequency of CD4^+^ and CD8^+^ T cells within the tumors and a significant decrease in the tumors of CD4+ CD25+FoxP3+ Tregs, of Arg1^+^ CD11b+ Gr1+ preMDSCs and of Arg1^+^ CD11b+ Ly6+ gramMDSCs indicating a negative impact of the treatment on the main immunosuppressive actors of the immune system. We also observed a significant increase in CXCR5^+^ B cells in vaccinated mice compared to controls suggesting that B-lymphocytes had migrated to tumor sites as a result of our treatment. We also have observed a significant increase in the frequency of CD44+ CD62L- CCR7low CD127low TEMs as well as in KLRG1 expression by TEMs in tumors suggesting that the immune memory had reverted to an active state, decreasing both anergy and senescence.

In our study, we did not observe any significant autoimmunity: immunized mice were generally healthy and presented no clinical evidence of autoimmune diseases. The animals' weight, hair, and musculature were normal. However, more follow-up is needed before iPS whole cell-based cancer vaccines move into clinical testing.

Taken together, our data show the feasibility of creating anti-cancer stem cell immunity through an approach that combines an HDACi and iPSC whole cell-based vaccine. These data strongly confirm that iPS cell-based vaccine + VPA in immune-competent mice have a broad mechanism action for metastatic aggressive tumors with stemness features. The principal mode of actions are driven by (i) enhanced trafficking with mobilized TILs in response to chemokines, (ii) improved cancer stem cell immune-recognition, (iii) decreased of MDSCs, (iv) recruited B cells into the tumor.

This technique can be easily applied to patients and, because it targets multiple TAAs from CSC-like cells and not from adult normal stem cells, this safe approach significantly decreases the occurrence of metastasis. This combinatory regimen should improve the overall survival of patients with stemness/ CSC-phenotype tumors associated with a dismal prognosis, particularly given that for solid tumors, invasion and metastasis account for more than 90% of mortality (65, 67). This new approach can be an alternative to the use of chemical compounds able to inhibit signaling pathways present in CSCs such as Stat3, mTor, Smo, Notch of Hedgehog inhibitors which are currently under study. Most of them have shown to be toxic and to cause collateral damages since all these signaling pathways are also heterogeneously expressed in the adult stem cell populations (68).

These beneficial properties of our approach make this combinatory regimen a potentially powerful option for a new concept of active immunotherapy that could be deployed shortly after conventional primary treatment of cancer or in combination with conventional therapies or “checkpoint inhibitors,” which are currently under intensive investigation.

## Data Availability Statement

The original contributions presented in the study are included in the article/Supplementary Material, further inquiries can be directed to the corresponding author.

## Ethics Statement

The animal study was reviewed and approved by Animal Care Committee of the Val de Marne.

## Author Contributions

FG, AB-G, and AT: direction and financing of the project. JA and MK: generation, production, and characterization of miPSCs. AA, MK, and FG: mouse experiments. MK and AA: molecular and cellular experiments and flow cytometry. CD: microarray and bioinformatic analysis. FG, AB-G, AT, MK, and AA: writing of the article.

## Funding

This work was performed with grants from ANR Programme d'Investissements d'Avenir of the INGESTEM National Infrastructure Program (ANR-11-INBS-0009-INGESTEM), Inserm, University Paris Saclay.

## Conflict of Interest

The authors declare that the research was conducted in the absence of any commercial or financial relationships that could be construed as a potential conflict of interest.

## Publisher's Note

All claims expressed in this article are solely those of the authors and do not necessarily represent those of their affiliated organizations, or those of the publisher, the editors and the reviewers. Any product that may be evaluated in this article, or claim that may be made by its manufacturer, is not guaranteed or endorsed by the publisher.

## Supplementary Material

Supplementary Figure 1 Development and ECM functional network up regulated in 4T1-transplant: **(A)** Barplot of functional enrichment performed on GOBP database (development and morphogenesis components). **(B)** Functional enrichment network implicated in developmental processes. **(C)** Barplot of functional enrichment performed on MSigDB database. **(D)** Functional enrichment network comprising relations with the microenvironment.

Supplementary Figure 2 Cell mobility and cell migration functional enriched network up regulated in 4T1-transplant. **(A)** Barplot of functional network performed on GOBP database. **(B)** Functional enrichment network implicated in cell migration and cell mobility.

Supplementary Figure 3 Quantification of CD44/CD24 markers in 4T1 cells by flow cytometry *in vitro* and *in vivo* 12 and 28 days after implantation into the fad pat of BALB/c mice.

Supplementary Figure 4 VPA treatment increased MHC I expression. 4T1 cells were incubated for 48 h with a dose of 0.2 and 2 mM VPA and MHC1 expression quantified by Flow cytometry using MHC Class I (H-2Kd/H-2Dd), eFluor 450 antibody. 4T1 MammoSpheres (MSs) were produced during 9 days in low-attachment 6-well plates at density of 100,000 cells per well in MEF-conditioned medium (3/4 MEF-conditioned medium + 1/4 mES medium + 4 ng/mL bFGF), and addition of TNF-alpha (20 ng/mL), and TGF-β 1 (10 ng/mL). **(A)** Increase in MHC I expression on the surface of 4T1 cells as a result of treatment with different doses of VPA (0, 0.2, and 2 mM), as revealed by flow cytometry. **(B)** Effect of treatment with 2 mM VPA on the expression of MHC I in adherent 4T1 cells and 4T1-derived MSs.

Supplementary Figure 5 Generation of MammoSpheres from the 4T1 cell line. 4T1 MammoSpheres (MSs) were produced in 3D culture condition using low-attachment 6-well-plates. 4T1 cells were cultured for 9 days at a density of 100,000 cells per well in MEF-conditioned medium (3/4 MEF-conditioned medium + 1/4 mES medium + 4 ng/mL bFGF), and addition of TNF-alpha (20 ng/mL), and TGF-β 1 (10 ng/mL). **(A)** Images of the morphology of adherent 4T1 cells and 4T1-derived MSs in 3D culture conditions, cultured with or without TGFβ + TNFα (magnification × 20). **(B)** Mammosphere-formation efficiency (calculated by Cell Selector Software) indicates the number of mammospheres of different sizes obtained with TGFβ + TNFα treatment. **(C)** Quantification of ALDH1 activity by flow cytometry in adherent 4T1 cells and in 4T1-derived mammospheres cultured with or without TGFβ + TNFα. **(D)** Percentage of ALDH1^+^ cells among adherent 4T1 cells and 4T1-derived mammospheres cultured with or without TGFβ + TNFα. Results are shown from three independent experiments.

Supplementary Figure 6 VPA-only treatment did not hinder *in vivo* tumor growth. Sixteen 8–10 weeks old females BALB/c mice, were divided into 2 groups: group control and mice treated with VPA orally administered at dose of 4 mg/m. All mice were inoculated with 5 × 10^4^ 4T1Luc cells into mammary fat-pad and tumors monitored for 21 days. **(A)** Tumor growth (mm^3^) in mice treated with VPA compared to control mice. **(B)** IVIS imaging of VPA-treated and untreated mice at day 21. **(C)** Lung metastases in VPA-treated and untreated mice were quantified using bioluminescence imaging. Regions of interest (ROI) for pulmonary metastases in these two groups were calculated by Living Image Software. **(D)** Effects of VPA treatment on the frequencies of CD4^+^ cells, CD8^+^ cells, Tregs, and MDSCs compared to controls, as quantified by flow cytometry.

Supplementary Figure 7 Vaccination and challenge protocol. Murine iPSCs were incubated for 24 h with 0.5 mM of VPA and irradiated at the dose of 15 Gy. Irradiated iPSCs were injected as a vaccine into BALB/c mice twice, with a one-week interval between doses. Mice were then challenged with 5 × 10^4^ 4T1 cells 1 week after the final dose. VPA was orally administered to vaccinated mice through their drinking water at a dose of 4 mg/mL.

Supplementary Figure 8 Characterization of murine induced pluripotent stem cells derived from BALB/c fibroblasts. Primary fibroblasts from BALB/c mice were reprogrammed into pluripotency by ectopic expression of OCT4, SOX2, c-MYC, and KLF4 using a Cre-Excisable Constitutive Polycistronic Lentivirus (EF1alpha-STEMCCA-LoxP backbone from Millipore). **(A)** Morphological view under the microscope of miPSCs expanded on mouse embryonic fibroblasts. **(B)** Expression of the key pluripotency markers NANOG and OCT4 as revealed by immunofluorescence; DAPI was used as counterstain. **(C)** Expression of SSEA1 in cell membranes as quantified by flow cytometry. **(D)** Teratoma formation assays, showing differentiation into ectodermal, endodermal, and mesodermal tissues.

Supplementary Figure 9 Tumor volumes of BALB/c mice treated with allogeneic C57BL/6-derived miPSCs + VPA compared to those of untreated mice. Eight to ten week old, females BALB/c mice were divided into 2 groups: group control (PBS; *n* = 7), and mice treated with miPSCs + VPA (*n* = 8). Treatment consisted of two sub-cutaneous injections (1-week interval between injections) of 2 × 10^6^ miPSCs. One week following the second injection, all mice were inoculated with 5 × 10^4^ 4T1Luc cells into mammary fat-pad following by 20 days of VPA treatment, orally administered at dose of 4 mg/m. The data represent the mean ± SEM of tumor volumes.

Supplementary Figure 10 Effective memory immune response following vaccination with miPSCs. Mice from treated group (*n* = 4) received 2 × 10^6^ irradiated BALB/c-derived miPSCs during 6 months, with a total of 6 injections, once every 30 days. Thirty days after the last vaccine inoculation, mice were challenged with 2.5 × 10^4^ 4T1Luc cells and vaccinated mice received 4 mg/mL VPA by oral route starting from the day of tumor injection until the sacrifice. Control mice (*n* = 4) received only PBS. **(A)** Experimental vaccination protocol evaluating induced *in vivo* cell memory immune response: BALB/c mice were injected subcutaneously six times with 2 × 10^6^ miPSCs (15 Gy irradiated) in the right flank. **(B)** At day 26 post-challenge, breast tumors were significantly smaller in mice that had undergone the 6-month vaccination protocol compared to unvaccinated mice (*n* = 5 per group). **(C)** Survival rate of mice treated by miPSC + VPA or by PBS (control group).

Supplementary Figure 11 Immune cell profiling of the spleens of mice treated with miPSCs + VPA **(A,B)** Frequency of CD4^+^ and CD8^+^ T cells in the spleens of mice treated with miPSCs+VPA compared with the control group, as measured by flow cytometry. **(C)** Frequency of PD1 expressed on cell membranes of CD4^+^ T cells, as measured by flow cytometry. **(D)** Frequency of PD1^+^ T-effector memory (CD44^+^CD62L^−^CCR7^low^CD127^low^) cells in the spleen, as measured by flow cytometry.

Supplementary Figure 12 Effective memory immune response following vaccination with miPSCs; Mice (*n* = 6) were treated with 2 × 10^6^ irradiated BALB/c-derived miPSCs during 6 months, with a total of 6 injections, once every 30 days. One hundred twenty days after the last vaccine inoculation, mice were challenged with 2.5 × 10^4^ 4T1Luc cells cultured in mammo sphere condition. Mammo sphere were performed from 4T1 that cultured for 9 days at density of 100,000 cells per well in MEF-conditioned medium (3/4 MEF-conditioned medium + 1/4 mES medium + 4 ng/mL bFGF), and addition of TNF-alpha (20 ng/mL), and TGF-β 1 (10 ng/mL). Mammo spheres were dissociated in PBS/EDTA before injection into both mammary fat-pad glands. Vaccinated mice received 4 mg/mL. VPA by oral route that was started from the day of tumor injection until the sacrifice. Control mice (*n* = 6) received only PBS. **(A)** Experimental protocol to evaluate *in vivo* immune memory generated by vaccination: BALB/c mice were injected subcutaneously six times with 2 × 10^6^ miPSCs (15 Gy irradiated) in the right flank. **(B)** At day 28 post-challenge, breast tumors were significantly smaller in mice that had undergone the 6-month vaccination protocol compared to unvaccinated mice. **(G)** Bioluminescence imaging of tumors from mice treated with miPSCs + VPA compared to untreated controls. **(H)** Bioluminescence imaging of lungs isolated from vaccinated and control mice 28 days after challenge. **(I)** A significant correlation was found between tumor burden and metastatic spread at day 28.

Supplementary Figure 13 CXCL9, CXCL10, and CXCL13 chemokines mRNA expressions in tumors from miPSC + VPA treated (*n* = 6) and control (*n* = 6) mice. Mice (*n* = 6) were treated with sub-cutaneous injections of 2 × 10^6^ irradiated BALB/c-derived miPSCs during 6 months, with a total of 6 injections, once every 30 days. One hundred twenty days after the last vaccine inoculation, mice were challenged with 2.5 × 10^4^ 4T1Luc cells and vaccinated mice received 4 mg/mL VPA by oral route starting from the day of tumor injection until the sacrifice. Control mice (*n* = 6) received only PBS. After 28 days RNAs of tumors were extracted for the quantification of CXCL9, CXCL10, and CXCL13 mRNA by real time RT-PCR.

Supplementary Table 1 List of antibodies; Names, references, and the dilution used.

Supplementary Table 2 Genes found up regulated in 4T1-transplant samples as compared to 4T1-*in vitro* done by LIMMA analysis: for each up regulated genes columns present respective statistics obtained by LIMMA algorithm: log_2_ Fold Change, average expression in microarray, *t*-statistics, *p*-value, False discovery rate adjusted *p*-values and *B*-statistics.

Supplementary Table 3 Differentially expressed immune genes that were upregulated in 4T1 cells by treatment with valproic acid. The list highlights immune-associated genes that were found to be significantly overexpressed in the treatment group using the Significance Analysis of Microarray algorithm with a false discovery rate of <5%. Columns include: gene symbol, gene description, gene ID number, and the fold-change in expression between the VPA-treated group and untreated controls.

Supplementary Table 4 Genes that were differentially expressed between vaccinated and control mice that developed 4T1-derived tumors. Differentially expressed genes are listed with their gene symbol, description, the fold-change in expression found in transcriptome experiments (Vaccine/Control), and the *p*-value of the comparison.

## References

[1] Dzobo K Senthebane DA Ganz C Thomford NE Wonkam A Dandara C. Advances in therapeutic targeting of cancer stem cells within the tumor microenvironment: an updated review. Cells. (2020) 9:1896. 10.3390/cells9081896 32823711 PMC7464860

[2] Kabakov A Yakimova A Matchuk O. Molecular chaperones in cancer stem cells: determinants of stemness and potential targets for antitumor therapy. Cells. (2020) 9:892. 10.3390/cells9040892 32268506 PMC7226806

[3] Nimmakayala RK Batra SK Ponnusamy MP. Unraveling the journey of cancer stem cells from origin to metastasis. Biochim Biophys Acta Rev Cancer. (2019) 1871:50–63. 10.1016/j.bbcan.2018.10.006 30419314 PMC6347501

[4] Shen YA Pan SC Chu I Lai RY Wei YH. Targeting cancer stem cells from a metabolic perspective. Exp Biol Med. (2020) 245:465–76. 10.1177/1535370220909309 32102562 PMC7082881

[5] Puram SV Tirosh I Parikh AS Patel AP Yizhak K Gillespie S. Single-Cell transcriptomic analysis of primary and metastatic tumor ecosystems in head and neck cancer. Cell. (2017) 171:1611–24. 10.1016/j.cell.2017.10.044 29198524 PMC5878932

[6] Galardi S Savino M Scagnoli F Pellegatta S Pisati F Zambelli F. Resetting cancer stem cell regulatory nodes upon MYC inhibition. EMBO Rep. (2016) 17:1872–89. 10.15252/embr.201541489 27852622 PMC5283599

[7] Malta TM Sokolov A Gentles AJ Burzykowski T Poisson L Weinstein JN. Machine learning identifies stemness features associated with oncogenic dedifferentiation. Cell. (2018) 173:338–54. 10.1016/j.cell.2018.03.034 29625051 PMC5902191

[8] Ben-Porath I Thomson MW Carey VJ Ge R Bell GW Regev A. An embryonic stem cell-like gene expression signature in poorly differentiated aggressive human tumors. Nat. Genet. (2008) 40:499–507. 10.1038/ng.127 18443585 PMC2912221

[9] Takahashi K Yamanaka S. Induction of pluripotent stem cells from mouse embryonic and adult fibroblast cultures by defined factors. Cell. (2006) 126:663–76. 10.1016/j.cell.2006.07.024 16904174

[10] Yu J Vodyanik MA Smuga-Otto K Antosiewicz-Bourget J Frane JL Tian S. Induced pluripotent stem cell lines derived from human somatic cells. Science. (2007) 318:1917–20. 10.1126/science.1151526 18029452

[11] Robinton DA Daley GQ. The promise of induced pluripotent stem cells in research and therapy. Nature. (2012) 481:295–305. 10.1038/nature10761 22258608 PMC3652331

[12] Hussein SM Batada NN Vuoristo S Ching RW Autio R Närvä E. Copy number variation and selection during reprogramming to pluripotency. Nature. (2011) 471:58–62. 10.1038/nature09871 21368824

[13] Schlaeger TM Daheron L Brickler TR Entwisle S Chan K Cianci A. A comparison of non-integrating reprogramming methods. Nat Biotechnol. (2015) 33:58–63. 10.1038/nbt.3070 25437882 PMC4329913

[14] Ouyang X Liu Y Zhou Y Guo J Wei TT Liu C Lee B. Antitumor effects of iPSC-based cancer vaccine in pancreatic cancer. Stem Cell Rep. (2021) 16:1468–77. 10.1016/j.stemcr.2021.04.004 33961792 PMC8190592

[15] Kooreman NG Kim Y de Almeida PE Termglinchan V Diecke S Shao NY. Autologous iPSC-Based vaccines elicit anti-tumor responses *in vivo*. Cell Stem Cell. (2018) 22:501–13. 10.1016/j.stem.2018.01.016 29456158 PMC6134179

[16] Li Y Zeng H Xu RH Liu B Li Z. Vaccination with human pluripotent stem cells generates a broad spectrum of immunological and clinical responses against colon cancer. Stem Cells. (2009) 27:3103–11. 10.1002/stem.234 19816950

[17] Yaddanapudi K Mitchell RA Putty K Willer S Sharma RK Yan J. Vaccination with embryonic stem cells protects against lung cancer: is a broad-spectrum prophylactic vaccine against cancer possible? PLoS ONE. (2012) 7:e42289. 10.1371/journal.pone.0042289 22860107 PMC3409174

[18] Zhang Z Chen X Chang X Ye X Li Y Cui H. Vaccination with embryonic stem cells generates effective antitumor immunity against ovarian cancer. Int J Mol Med. (2013) 31:147–53. 10.3892/ijmm.2012.1195 23174760

[19] Miranda A Hamilton PT Zhang AW Pattnaik S Becht E Mezheyeuski A. Cancer stemness, intratumoral heterogeneity, and immune response across cancers. Proc Natl Acad Sci USA. (2019) 116:9020–9. 10.1073/pnas.1818210116 30996127 PMC6500180

[20] Yang Y Wang Y. Role of epigenetic regulation in plasticity of tumor immune. Microenvironment. (2021) 2:640369. 10.3389/fimmu.2021.640369 33868269 PMC8051582

[21] Topper MJ Vaz M Marrone KA Brahmer JR Baylin SB. The emerging role of epigenetic therapeutics in immuno-oncology. Nat Rev Clin Oncol. (2020) 17:75–90. 10.1038/s41571-019-0266-5 31548600 PMC7254932

[22] Xie Z Ikegami T Ago Y Okada N Tachibana M. Valproic acid attenuates CCR2-dependent tumor infiltration of monocytic myeloid-derived suppressor cells, limiting tumor progression. Oncoimmunology. (2020) 9:1734268. 10.1080/2162402X.2020.1734268 32158627 PMC7051186

[23] Terranova-Barberio M Serena Roca M Zotti AI Leone A Bruzzese F Vitagliano C. Valproic acid potentiates the anticancer activity of capecitabine *in vitro* and *in vivo* in breast cancer models via induction of thymidine phosphorylase expression. Oncotarget. (2016) 7:7715–7731. 10.18632/oncotarget.6802 26735339 PMC4884949

[24] Griscelli F Féraud O Oudrhiri N Gobbo E Casal I Chomel JC. Malignant germ cell-like tumors, expressing Ki-1 antigen (CD30), are revealed during *in vivo* differentiation of partially reprogrammed human-induced pluripotent stem cells. Am J Pathol. (2012) 180:2084–96. 10.1016/j.ajpath.2012.01.011 22425713

[25] Huangfu D Osafune K Maehr R Guo W Eijkelenboom A Chen S. Induction of pluripotent stem cells from primary human fibroblasts with only Oct4 and Sox2. Nat Biotechnol. (2008) 26:1269–75. 10.1038/nbt.1502 18849973

[26] Chen X Zhai Y Yu D Cui J Hu JF Li W. Valproic acid enhances iPSC induction from human bone marrow-derived cells through the suppression of reprogramming-induced senescence. J Cell Physiol. (2016) 231:1719–27. 10.1002/jcp.25270 26620855

[27] Leek JT Johnson WE Parker HS Jaffe AE Storey JD. The sva package for removing batch effects and other unwanted variation in high-throughput experiments. Bioinformatics. (2012) 28:882–3. 10.1093/bioinformatics/bts034 22257669 PMC3307112

[28] Ritchie ME Phipson B Wu D Hu Y Law CW Shi W. Limma powers differential expression analyses for RNA-sequencing and microarray studies. Nucleic Acids Res. (2015) 43:e47. 10.1093/nar/gkv007 25605792 PMC4402510

[29] Chen J Bardes EE Bruce J Aronow BJ Jegga AG. ToppGene suite for gene list enrichment analysis and candidate gene prioritization. Nucleic Acids Res. (2009) 37:W305–311. 10.1093/nar/gkp427 19465376 PMC2703978

[30] Liberzon A Birger C Thorvaldsdóttir H Ghandi M Mesirov JP Pablo Tamayo P. The molecular signatures database (MSigDB) hallmark gene set collection. Cell Systems. (2015) 6:417–25. 10.1016/j.cels.2015.12.004 26771021 PMC4707969

[31] Cline MS Smoot M Cerami E Kuchinsky A Landys N Workman C. Integration of biological networks and gene expression data using cytoscape. Nat. Protoc. (2007) 2:2366–82. 10.1038/nprot.2007.324 17947979 PMC3685583

[32] Shabbeer S Kortenhorst MS Kachhap S Galloway N Rodriguez R Carducci MA. Multiple molecular pathways explain the anti-proliferative effect of valproic acid on prostate cancer cells *in vitro* and *in vivo*. Prostate. (2007) 67:1099–110. 10.1002/pros.20587 17477369

[33] Pearce DJ Taussig D Simpson C Allen K Rohatiner AZ Lister TA. Characterization of cells with a high aldehyde dehydrogenase activity from cord blood and acute myeloid leukemia samples. Stem Cells. (2005) 23:752–60. 10.1634/stemcells.2004-0292 15917471

[34] Irizarry RA Bolstad BM Collin F Cope LM Hobbs B Speed TP. Summaries of affymetrix genechip probe level data. Nucleic Acids Res. (2003) 31:e15. 10.1093/nar/gng015 12582260 PMC150247

[35] Lyons YA Wu SY Overwijk WW Baggerly KA Sood AK. Immune cell profiling in cancer: molecular approaches to cell-specific identification. NPJ Precis Oncol. (2017) 1:26. 10.1038/s41698-017-0031-0 29872708 PMC5871917

[36] Subramanian A Tamayo P Mootha VK Mukherjee S Ebert BL Gillette MA. Gene set enrichment analysis: a knowledge-based approach for interpreting genome-wide expression profiles. Proc Natl Acad Sci USA. (2005) 102:15545–50. 10.1073/pnas.0506580102 16199517 PMC1239896

[37] Huang DW Sherman BT Lempicki RA. Systematic and integrative analysis of large gene lists using DAVID bioinformatics resources. Nat Protoc. (2009) 4:44–57. 10.1038/nprot.2008.211 19131956

[38] Mani SA Guo W Liao MJ Eaton EN Ayyanan A Zhou AY. The epithelial-mesenchymal transition generates cells with properties of stem cells. Cells. (2008) 133:704–15. 10.1016/j.cell.2008.03.027 18485877 PMC2728032

[39] Sigalotti L Fratta E Coral S Maio M. Epigenetic drugs as immunomodulators for combination therapies in solid tumors. Pharmacol Ther. (2014) 142:339–50. 10.1016/j.pharmthera.2013.12.015 24384533

[40] Dunn J Rao S. Epigenetics and immunotherapy: the current state of play. Mol Immunol. (2017) 87:227–39. 10.1016/j.molimm.2017.04.012 28511092

[41] De Almeida PE Meyer EH Kooreman NG Diecke S Dey D Sanchez- Freire V. Transplanted terminally differentiated induced pluripotent stem cells are accepted by immune mechanisms similar to self-tolerance. Nat Commun. (2014) 5:3903. 10.1038/ncomms4903 24875164 PMC4075468

[42] Zhao T Zhang ZN Rong Z Xu Y. Immunogenicity of induced pluripotent stem cells. Nature. (2011) 474:212–15. 10.1038/nature10135 21572395

[43] Yao Y Chu Y Xu B Hu Q Qibin Song Q. Risk factors for distant metastasis of patients with primary triple-negative breast cancer. Biosci Rep. (2019) 39:BSR20190288. 10.1042/BSR20190288 31113872 PMC6549086

[44] Wagenblast E Soto M Gutierrez-Angel S Hartl CA Gable AL Maceli AR. A model of breast cancer heterogeneity reveals vascular mimicry as a driver of metastasis. Nature. (2015) 520:358–62. 10.1038/nature14403 25855289 PMC4634366

[45] Prager BC Xie Q Bao S Rich JN. Cancer stem cells: the architects of the tumor ecosystem. Cell Stem Cell. (2019) 24:41–53. 10.1016/j.stem.2018.12.009 30609398 PMC6350931

[46] Marks PA Xu WS. Histone deacetylase inhibitors: potential in cancer therapy. J Cell Biochem. (2009) 1:600–8. 10.1002/jcb.22185 19459166 PMC2766855

[47] Fratta E Coral S Covre A Parisi G Colizzi F Danielli R. The biology of cancer testis antigens: putative function, regulation and therapeutic potential. Mol Oncol. (2011) 5:164–82. 10.1016/j.molonc.2011.02.001 21376678 PMC5528287

[48] Murakami T Sato A Chun NAL Hara M Naito Y Kobayashi Y. Transcriptional modulation using HDACi depsipeptide promotes immune cell-mediated tumor destruction of murine B16 melanoma. J Invest Dermatol. (2008) 128:1506–16. 10.1038/sj.jid.5701216 18185535

[49] De Lourdes Mora-García M Duenas-González A Hernández-Montes J De la Cruz-Hernández E Pérez-Cárdenas E Weiss-Steider B. Up-regulation of HLA class-I antigen expression and antigen-specific CTL response in cervical cancer cells by the demethylating agent hydralazine and the histone deacetylase inhibitor valproic acid. J Transl Med. (2006) 27:55. 10.1186/1479-5876-4-55 17192185 PMC1781077

[50] Armeanu S Bitzer M Lauer UM Venturelli S Pathil A Krusch M. Natural killer cell-mediated lysis of hepatoma cells via specific induction of NKG2D ligands by the histone deacetylase inhibitor sodium valproate. Cancer Res. (2005) 65:6321–9. 10.1158/0008-5472.CAN-04-4252 16024634

[51] Sahakian E Powers JJ Chen J Deng SL Cheng F Distler A. Histone deacetylase 11: a novel epigenetic regulator of myeloid derived suppressor cell expansion and function. Mol Immunol. (2015) 63:579–85. 10.1016/j.molimm.2014.08.002 25155994 PMC4252813

[52] Zheng H Zhao W Yan C Watson CC Massengill M Xie M. HDAC inhibitors enhance t-cell chemokine expression and augment response to PD-1 immunotherapy in lung adenocarcinoma. Clin Cancer Res. (2016) 22:4119–32. 10.1158/1078-0432.CCR-15-2584 26964571 PMC4987196

[53] Zhu S Denman CJ Cobanoglu ZS Kiany S Lau CC Gottschalk SM Hughes DP. The narrow-spectrum HDAC inhibitor entinostat enhances NKG2D expression without NK cell toxicity, leading to enhanced recognition of cancer cells. Pharm Res. (2015) 32:779–92. 10.1007/s11095-013-1231-0 24203492 PMC4014531

[54] Yang D Torres CM Bardhan K Zimmerman M McGaha TL Liu K. Decitabine and vorinostat cooperate to sensitize colon carcinoma cells to Fas ligand-induced apoptosis in vitro and tumor suppression *in vivo*. J Immunol. (2012) 188:4441–9. 10.4049/jimmunol.1103035 22461695 PMC3398838

[55] Hao Q Vadgama JV Wang P. CCL2/CCR2 signaling in cancer pathogenesis. Cell Commun Signal. (2020) 18:82. 10.1186/s12964-020-00589-8 32471499 PMC7257158

[56] Wang HF Ning F Liu ZC Wu L Li ZQ Qi YF. Histone deacetylase inhibitors deplete myeloid-derived suppressor cells induced by 4T1 mammary tumors *in vivo* and in vitro. Cancer Immunol Immunother. (2017) 66:355–66. 10.1007/s00262-016-1935-1 27915371 PMC11028551

[57] Riester M Wu HJ Zehir A Gonen M Moreira AL Downey RJ Michor F. Distance in cancer gene expression from stem cells predicts patient survival. (2017) 12:e0173589. 10.1371/journal.pone.0173589 28333954 PMC5363813

[58] Creighton CJ Li X Landis M Dixon JM Neumeister VM Sjolund A. Residual breast cancers after conventional therapy display mesenchymal as well as tumor-initiating features. Proc Natl Acad Sci USA. (2009) 106:13820–5. 10.1073/pnas.0905718106 19666588 PMC2720409

[59] Shafee N Smith CR Wei S Kim Y Mills GB Hortobagyi GN. Cancer stem cells contribute to cisplatin resistance in Brca1/p53-mediated mouse mammary tumors. Cancer Res. (2008) 68:3243–50. 10.1158/0008-5472.CAN-07-5480 18451150 PMC2929908

[60] Yamauchi K Yang M Hayashi K Jiang P Yamamoto N Tsuchiya H. Induction of cancer metastasis by cyclophosphamide pretreatment of host mice: an opposite effect of chemotherapy. Cancer Res. (2008) 68:516–20. 10.1158/0008-5472.CAN-07-3063 18199547

[61] Plaks V Kong N Werb Z. The cancer stem cell niche: how essential is the niche in regulating stemness of tumor cells? Cell Stem Cell. (2015) 16:225–38. 10.1016/j.stem.2015.02.015 25748930 PMC4355577

[62] Holohan C Van Schaeybroeck S Longley DB Johnston PG. Cancer drug resistance: an evolving paradigm. Nat Rev Cancer. (2013) 13:714–26. 10.1038/nrc3599 24060863

[63] Diehn M Cho RW Lobo NA Kalisky T Dorie MJ Kulp AN. Association of reactive oxygen species levels and radioresistance in cancer stem cells. Nature. (2009) 458:780–3. 10.1038/nature07733 19194462 PMC2778612

[64] Bao S Wu Q McLendon RE Hao Y Shi Q Hjelmeland AB. Glioma stem cells promote radioresistance by preferential activation of the DNA damage response. Nature. (2006) 444:756–60. 10.1038/nature05236 17051156

[65] Ganesh K Massagué J. Targeting metastatic cancer. Nat Med. (2021) 27:34. 10.1038/s41591-020-01195-4 33442008 PMC7895475

[66] Schoenhals M Kassambara A De Vos J Hose D Moreaux J Klein B. Embryonic stem cell markers expression in cancers. Biochem Biophys Res Commun. (2009) 383:157–62. 10.1016/j.bbrc.2009.02.156 19268426

[67] Venetis K Piciotti R Sajjadi E Invernizzi M Morganti S Criscitiello C. Breast cancer with bone metastasis: molecular insights and clinical management. Cells. (2021) 10:1377. 10.3390/cells10061377 34199522 PMC8229615

[68] Yang L Shi P Zhao G Xu J Peng W Zhang J. Targeting cancer stem cell pathways for cancer therapy. Signal Transduct Target Ther. (2020) 5:8. 10.1038/s41392-020-0110-5 32296030 PMC7005297

