# Supplementary figures and images for: Evidence of Antitumor and Antimetastatic Potential of Induced Pluripotent Stem Cell-Based Vaccines in Cancer Immunotherapy
Source: Front Med (Lausanne). 2021 Dec 10;8:729018. doi: 10.3389/fmed.2021.729018 (PMC8702815; doi:10.3389/fmed.2021.729018)

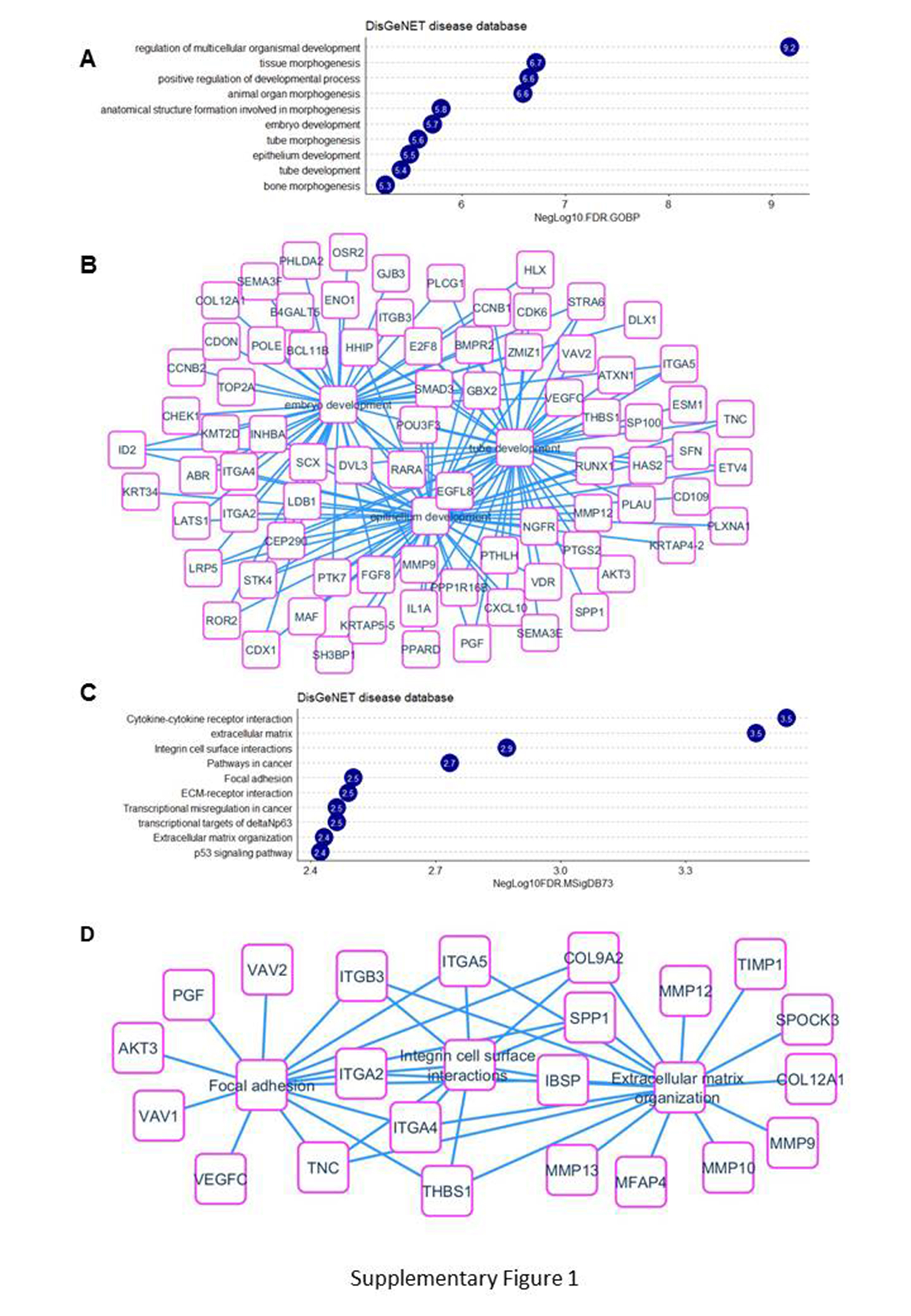

Supplement: Supplementary Figure 1 — Development and ECM functional network up regulated in 4T1-transplant: (A) Barplot of functional enrichment performed on GOBP database (development and morphogenesis components). (B) Functional enrichment network implicated in developmental processes. (C) Barplot of functional enrichment performed on MSigDB database. (D) Functional enrichment network comprising relations with the microenvironment. [file Image_1.JPEG]

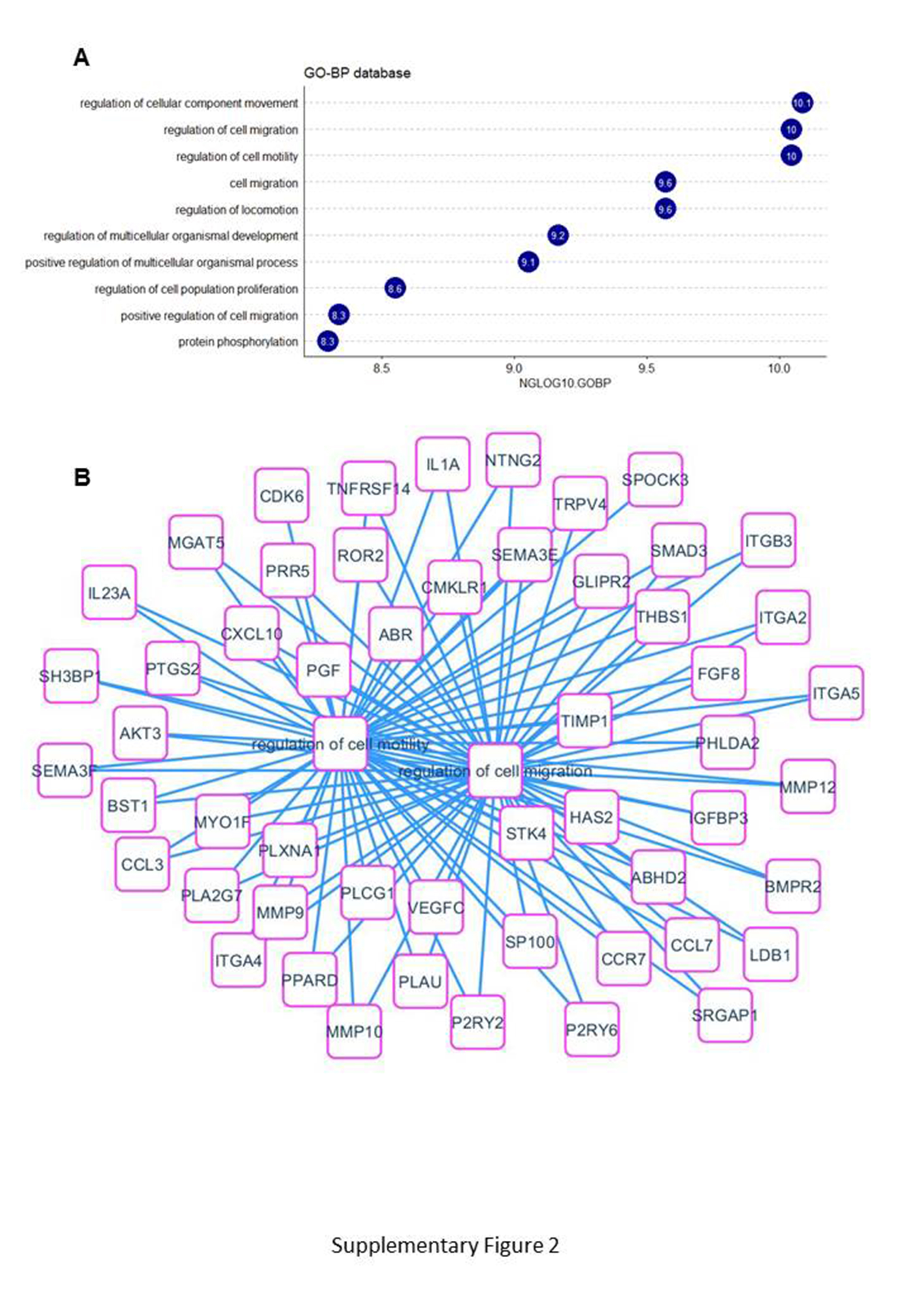

Supplement: Supplementary Figure 2 — Cell mobility and cell migration functional enriched network up regulated in 4T1-transplant. (A) Barplot of functional network performed on GOBP database. (B) Functional enrichment network implicated in cell migration and cell mobility. [file Image_2.JPEG]

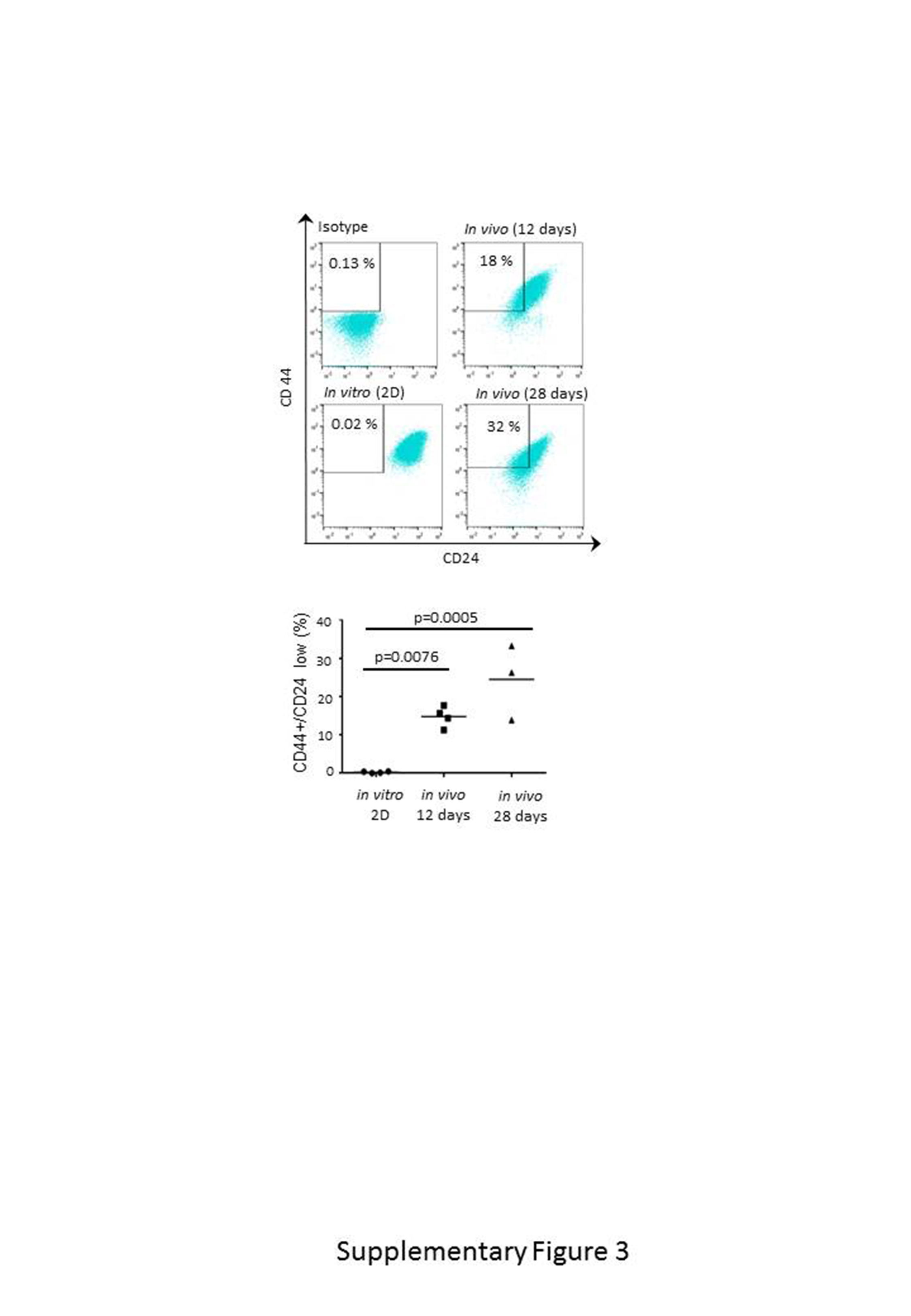

Supplement: Supplementary Figure 3 — Quantification of CD44/CD24 markers in 4T1 cells by flow cytometry in vitro and in vivo 12 and 28 days after implantation into the fad pat of BALB/c mice. [file Image_3.JPEG]

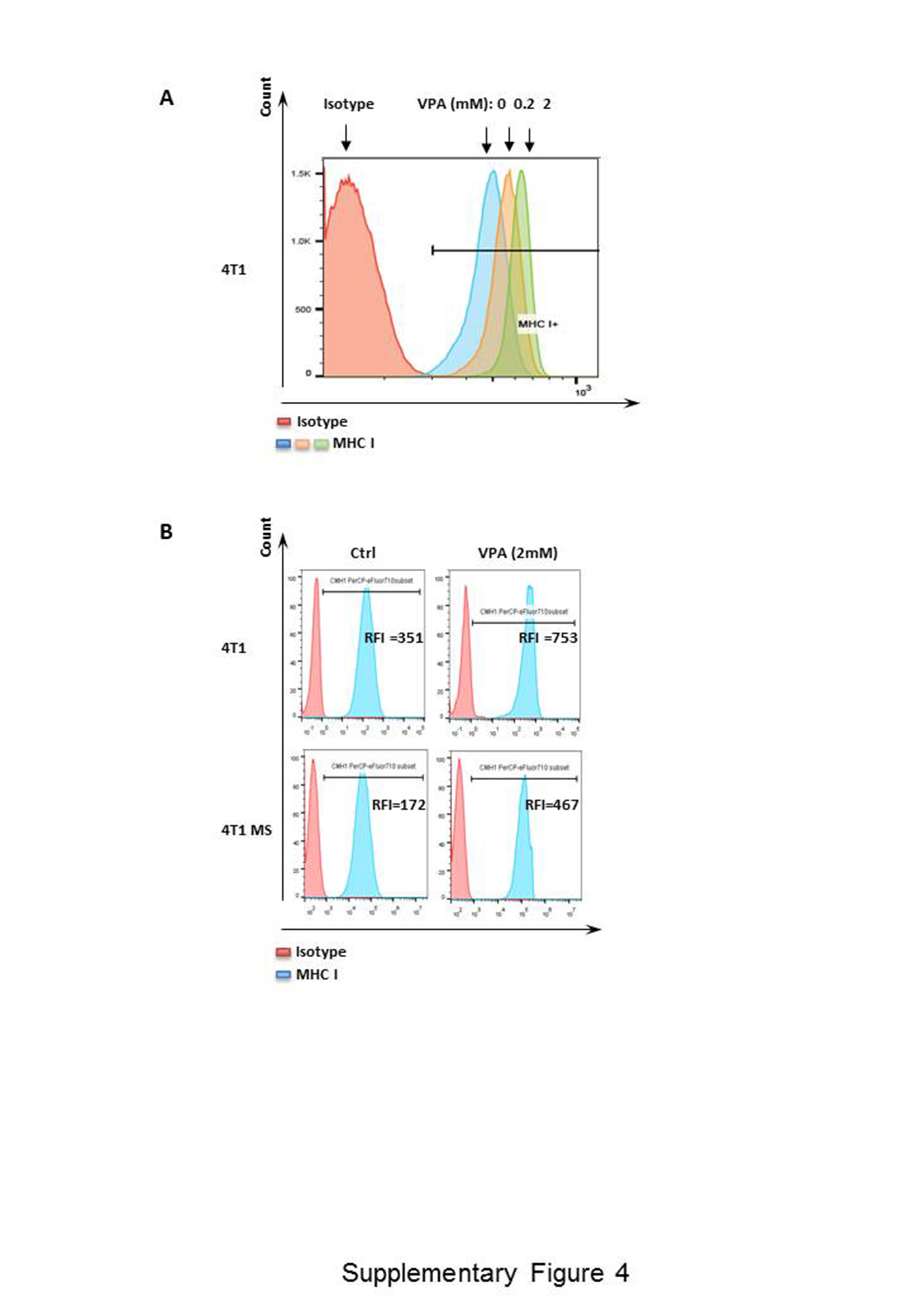

Supplement: Supplementary Figure 4 — VPA treatment increased MHC I expression. 4T1 cells were incubated for 48 h with a dose of 0.2 and 2 mM VPA and MHC1 expression quantified by Flow cytometry using MHC Class I (H-2Kd/H-2Dd), eFluor 450 antibody. 4T1 MammoSpheres (MSs) were produced during 9 days in low-attachment 6-well plates at density of 100,000 cells per well in MEF-conditioned medium (3/4 MEF-conditioned medium + 1/4 mES medium + 4 ng/mL bFGF), and addition of TNF-alpha (20 ng/mL), and TGF-β 1 (10 ng/mL). (A) Increase in MHC I expression on the surface of 4T1 cells as a result of treatment with different doses of VPA (0, 0.2, and 2 mM), as revealed by flow cytometry. (B) Effect of treatment with 2 mM VPA on the expression of MHC I in adherent 4T1 cells and 4T1-derived MSs. [file Image_4.JPEG]

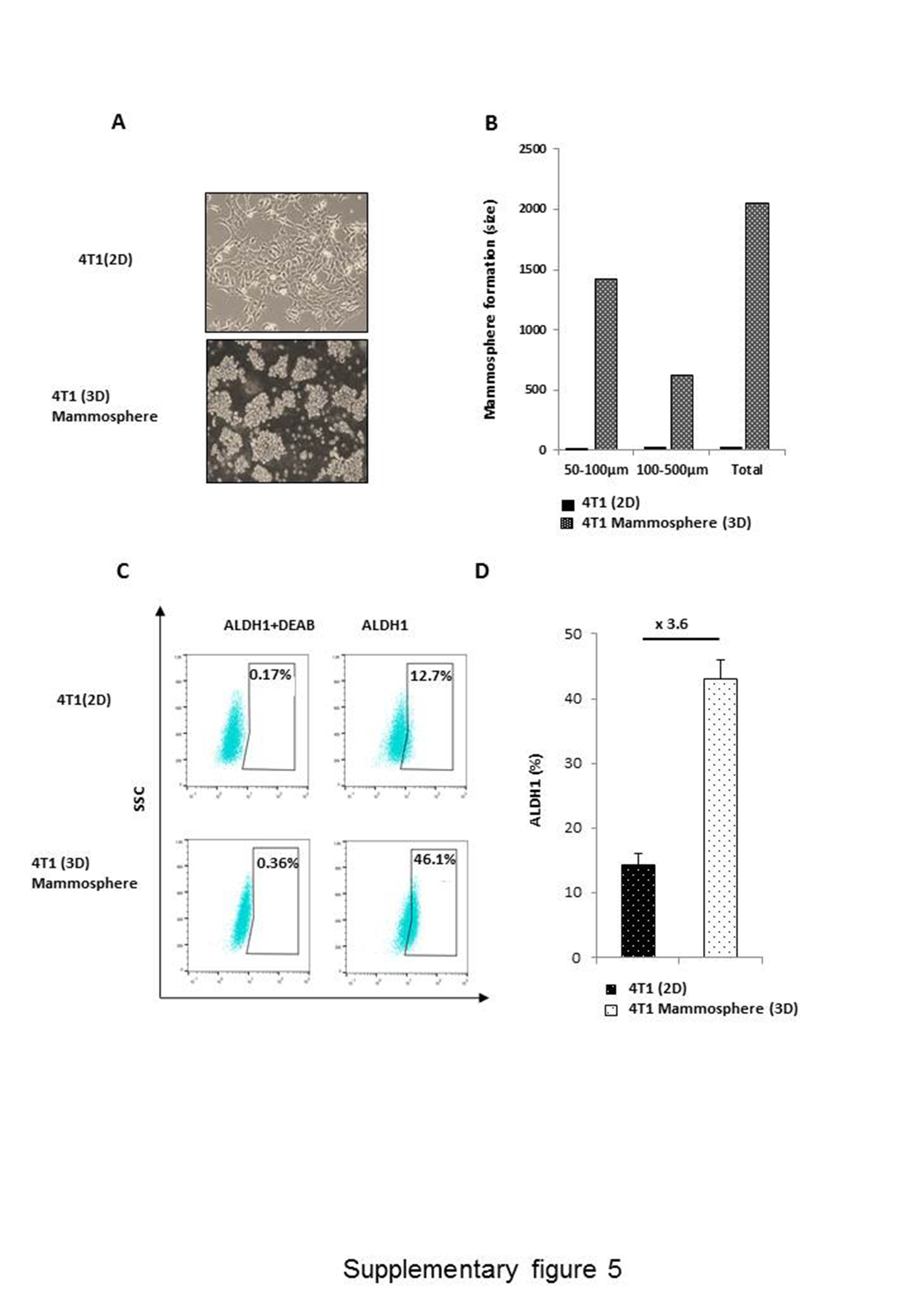

Supplement: Supplementary Figure 5 — Generation of MammoSpheres from the 4T1 cell line. 4T1 MammoSpheres (MSs) were produced in 3D culture condition using low-attachment 6-well-plates. 4T1 cells were cultured for 9 days at a density of 100,000 cells per well in MEF-conditioned medium (3/4 MEF-conditioned medium + 1/4 mES medium + 4 ng/mL bFGF), and addition of TNF-alpha (20 ng/mL), and TGF-β 1 (10 ng/mL). (A) Images of the morphology of adherent 4T1 cells and 4T1-derived MSs in 3D culture conditions, cultured with or without TGFβ + TNFα (magnification × 20). (B) Mammosphere-formation efficiency (calculated by Cell Selector Software) indicates the number of mammospheres of different sizes obtained with TGFβ + TNFα treatment. (C) Quantification of ALDH1 activity by flow cytometry in adherent 4T1 cells and in 4T1-derived mammospheres cultured with or without TGFβ + TNFα. (D) Percentage of ALDH1+ cells among adherent 4T1 cells and 4T1-derived mammospheres cultured with or without TGFβ + TNFα. Results are shown from three independent experiments. [file Image_5.JPEG]

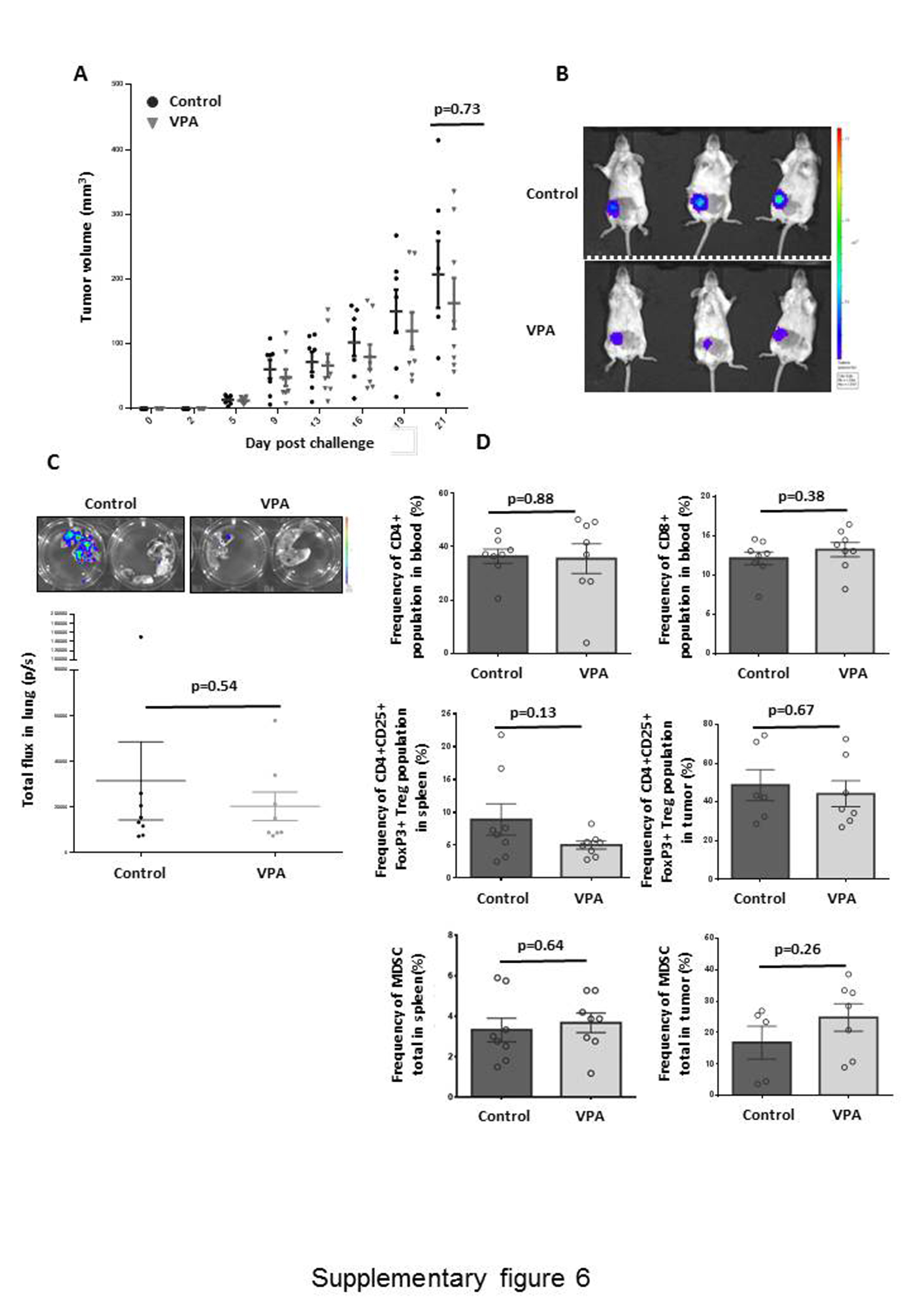

Supplement: Supplementary Figure 6 — VPA-only treatment did not hinder in vivo tumor growth. Sixteen 8–10 weeks old females BALB/c mice, were divided into 2 groups: group control and mice treated with VPA orally administered at dose of 4 mg/m. All mice were inoculated with 5 × 10^4^ 4T1Luc cells into mammary fat-pad and tumors monitored for 21 days. (A) Tumor growth (mm3) in mice treated with VPA compared to control mice. (B) IVIS imaging of VPA-treated and untreated mice at day 21. (C) Lung metastases in VPA-treated and untreated mice were quantified using bioluminescence imaging. Regions of interest (ROI) for pulmonary metastases in these two groups were calculated by Living Image Software. (D) Effects of VPA treatment on the frequencies of CD4+ cells, CD8+ cells, Tregs, and MDSCs compared to controls, as quantified by flow cytometry. [file Image_6.JPEG]

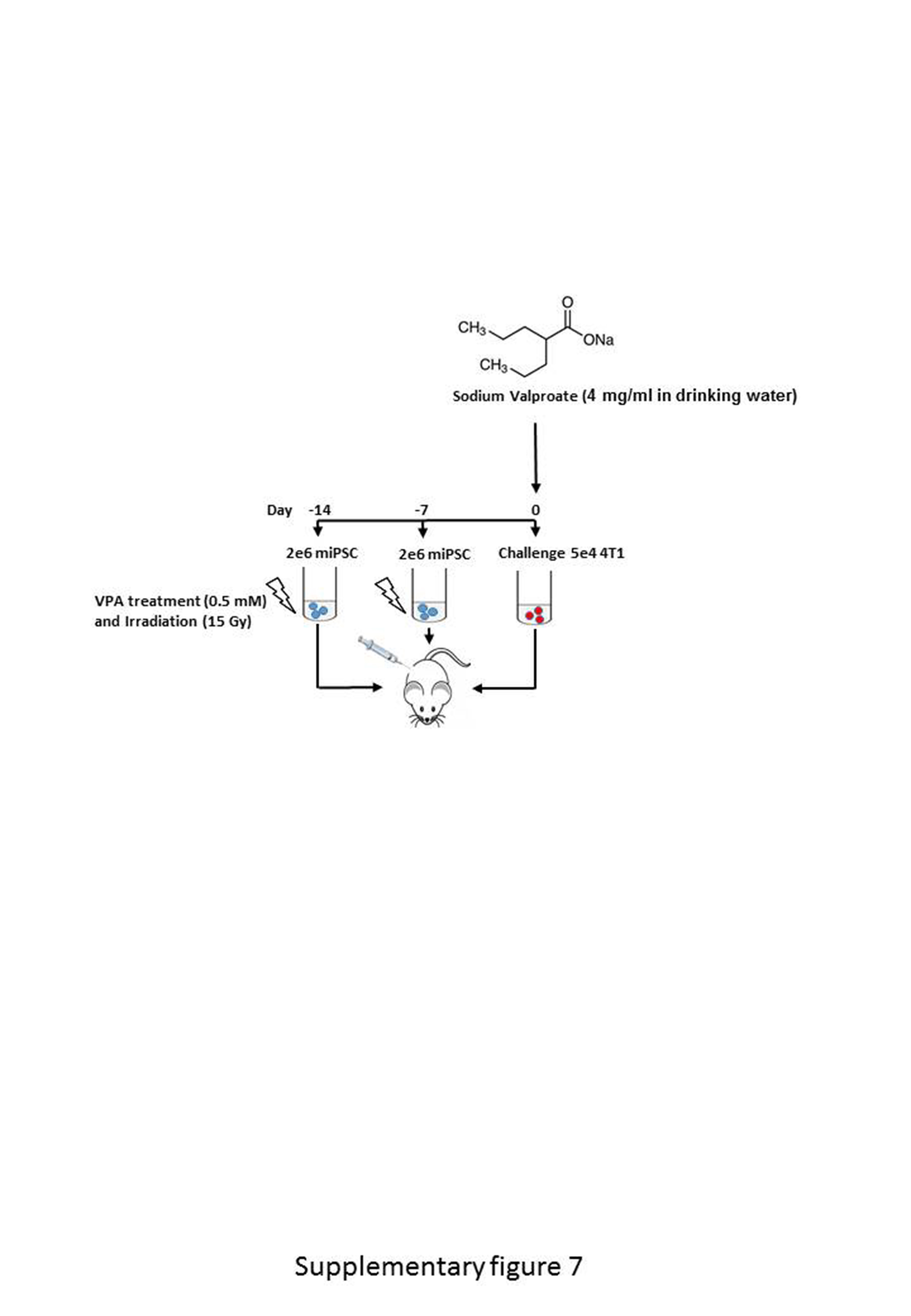

Supplement: Supplementary Figure 7 — Vaccination and challenge protocol. Murine iPSCs were incubated for 24 h with 0.5 mM of VPA and irradiated at the dose of 15 Gy. Irradiated iPSCs were injected as a vaccine into BALB/c mice twice, with a one-week interval between doses. Mice were then challenged with 5 × 10^4^ 4T1 cells 1 week after the final dose. VPA was orally administered to vaccinated mice through their drinking water at a dose of 4 mg/mL. [file Image_7.JPEG]

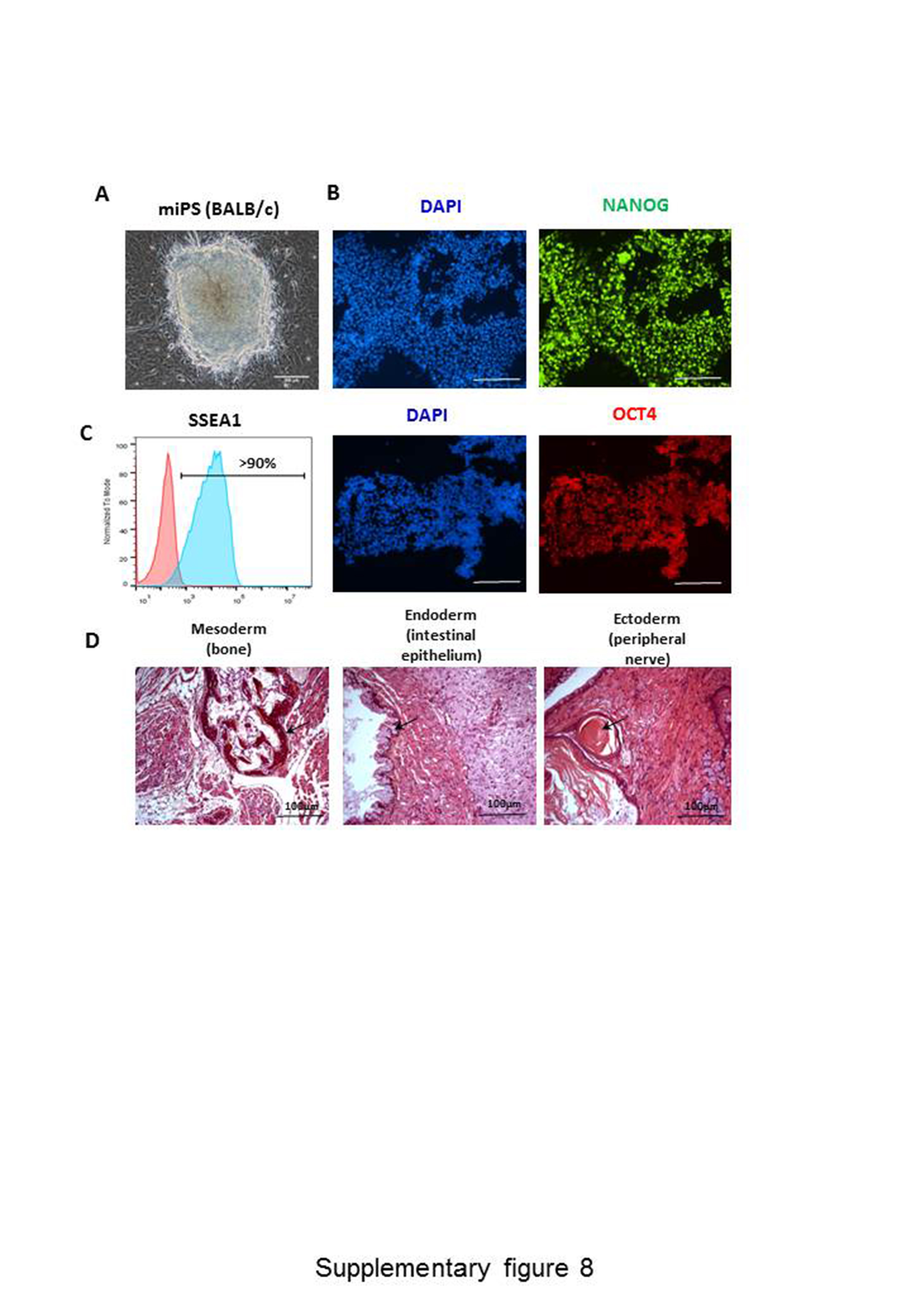

Supplement: Supplementary Figure 8 — Characterization of murine induced pluripotent stem cells derived from BALB/c fibroblasts. Primary fibroblasts from BALB/c mice were reprogrammed into pluripotency by ectopic expression of OCT4, SOX2, c-MYC, and KLF4 using a Cre-Excisable Constitutive Polycistronic Lentivirus (EF1alpha-STEMCCA-LoxP backbone from Millipore). (A) Morphological view under the microscope of miPSCs expanded on mouse embryonic fibroblasts. (B) Expression of the key pluripotency markers NANOG and OCT4 as revealed by immunofluorescence; DAPI was used as counterstain. (C) Expression of SSEA1 in cell membranes as quantified by flow cytometry. (D) Teratoma formation assays, showing differentiation into ectodermal, endodermal, and mesodermal tissues. [file Image_8.JPEG]

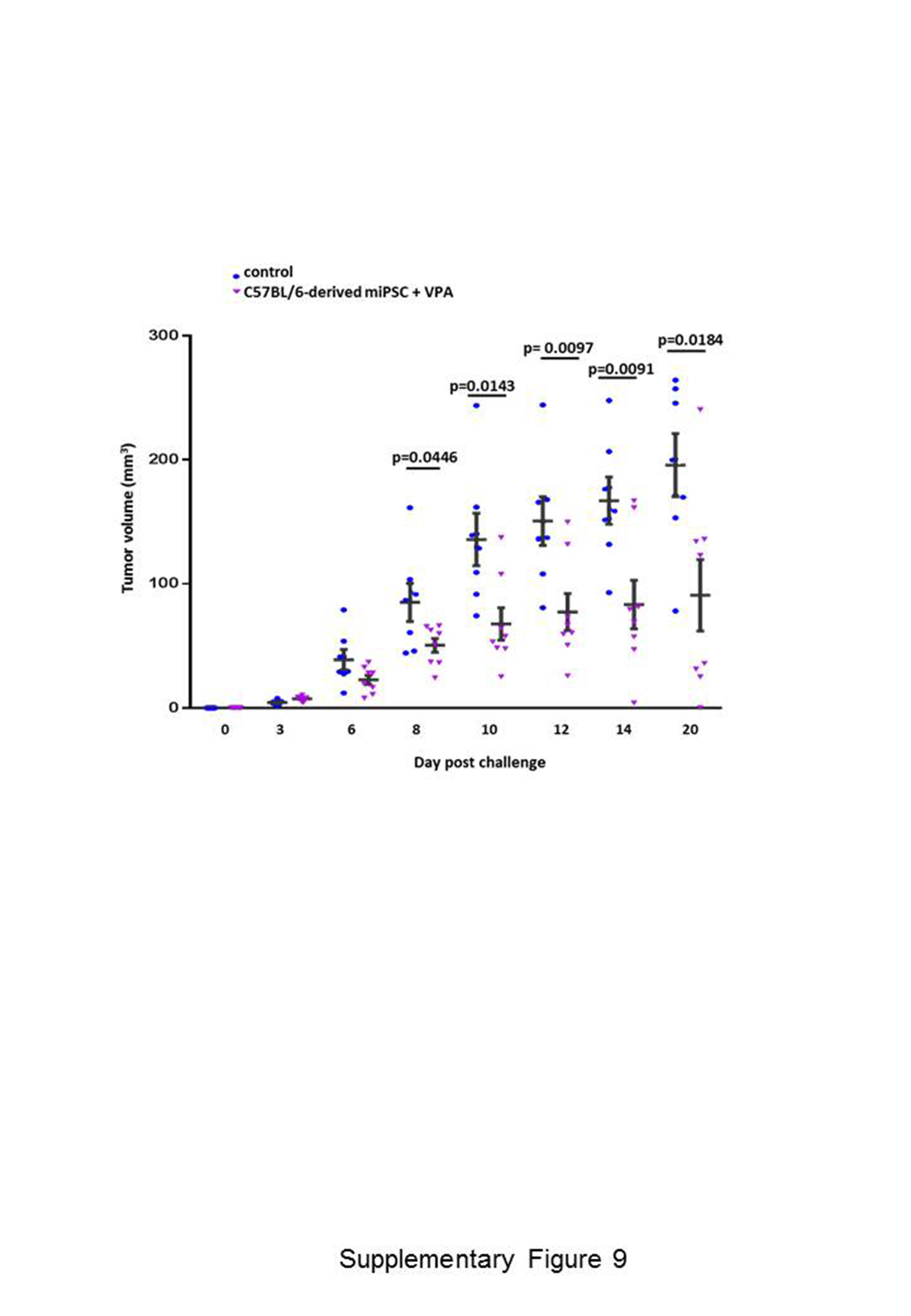

Supplement: Supplementary Figure 9 — Tumor volumes of BALB/c mice treated with allogeneic C57BL/6-derived miPSCs + VPA compared to those of untreated mice. Eight to ten week old, females BALB/c mice were divided into 2 groups: group control (PBS; n = 7), and mice treated with miPSCs + VPA (n = 8). Treatment consisted of two sub-cutaneous injections (1-week interval between injections) of 2 × 10^6^ miPSCs. One week following the second injection, all mice were inoculated with 5 × 10^4^ 4T1Luc cells into mammary fat-pad following by 20 days of VPA treatment, orally administered at dose of 4 mg/m. The data represent the mean ± SEM of tumor volumes. [file Image_9.JPEG]

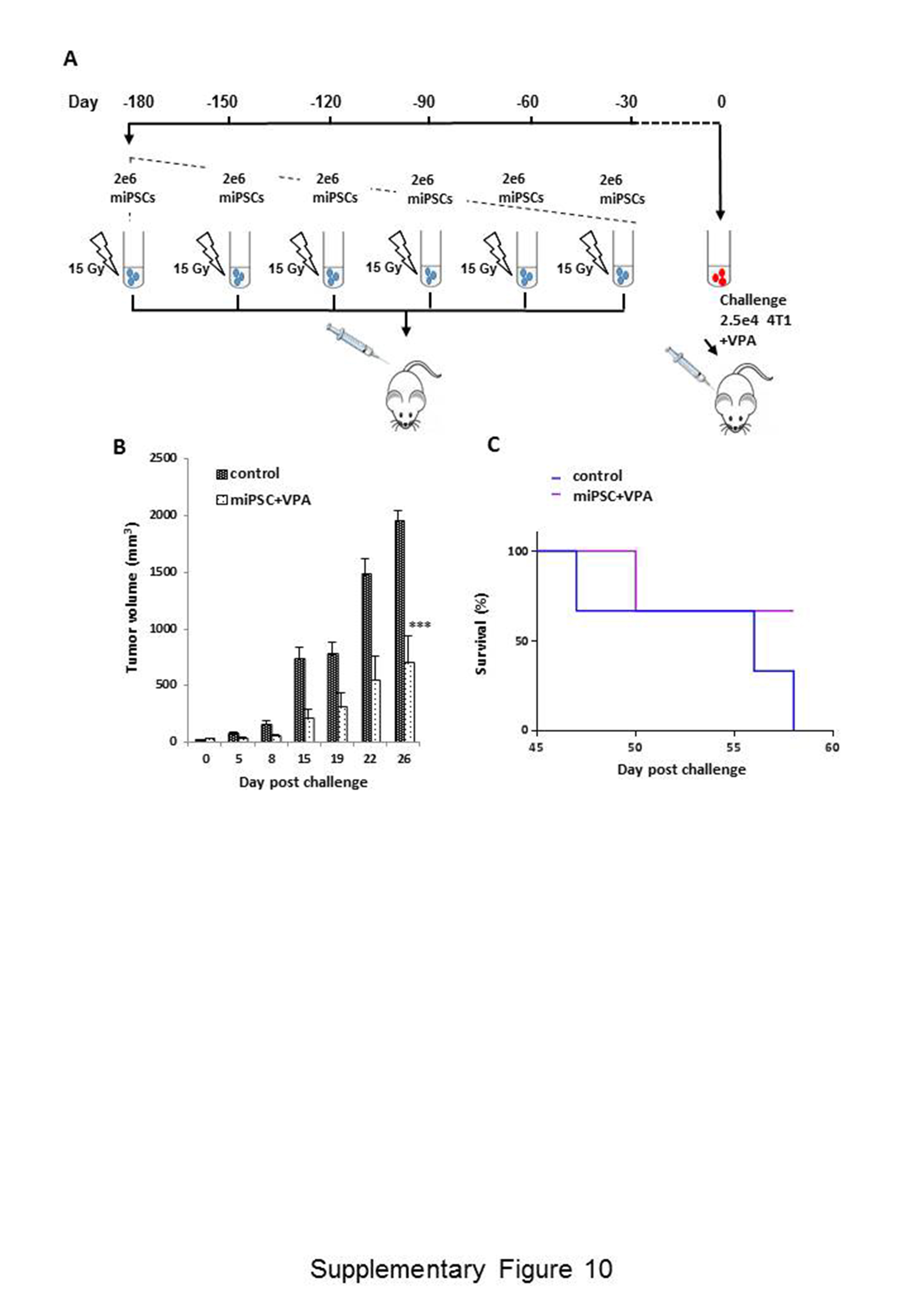

Supplement: Supplementary Figure 10 — Effective memory immune response following vaccination with miPSCs. Mice from treated group (n = 4) received 2 × 10^6^ irradiated BALB/c-derived miPSCs during 6 months, with a total of 6 injections, once every 30 days. Thirty days after the last vaccine inoculation, mice were challenged with 2.5 × 10^4^ 4T1Luc cells and vaccinated mice received 4 mg/mL VPA by oral route starting from the day of tumor injection until the sacrifice. Control mice (n = 4) received only PBS. (A) Experimental vaccination protocol evaluating induced in vivo cell memory immune response: BALB/c mice were injected subcutaneously six times with 2 × 10^6^ miPSCs (15 Gy irradiated) in the right flank. (B) At day 26 post-challenge, breast tumors were significantly smaller in mice that had undergone the 6-month vaccination protocol compared to unvaccinated mice (n = 5 per group). (C) Survival rate of mice treated by miPSC + VPA or by PBS (control group). [file Image_10.JPEG]

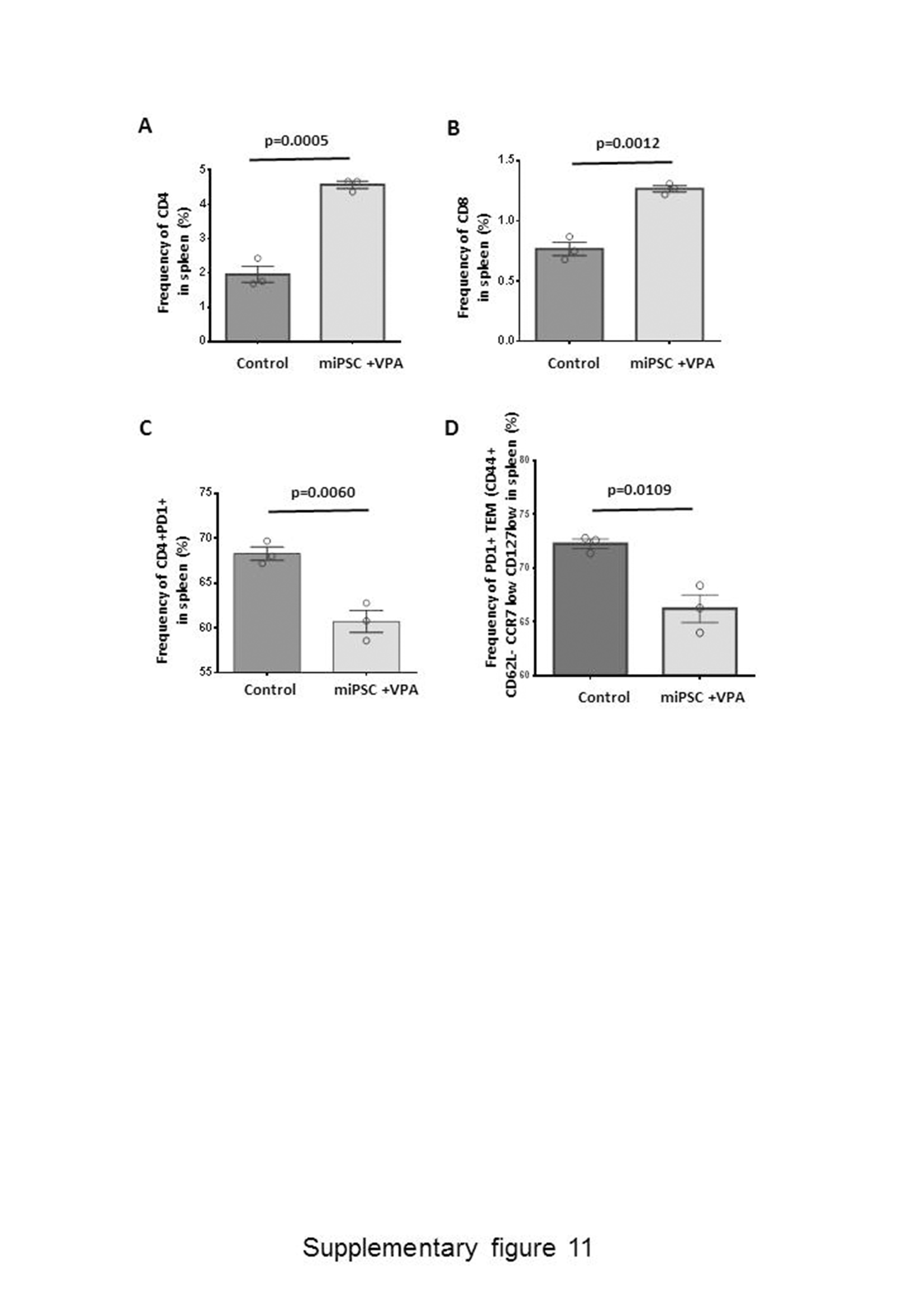

Supplement: Supplementary Figure 11 — Immune cell profiling of the spleens of mice treated with miPSCs + VPA (A,B) Frequency of CD4+ and CD8+ T cells in the spleens of mice treated with miPSCs+VPA compared with the control group, as measured by flow cytometry. (C) Frequency of PD1 expressed on cell membranes of CD4+ T cells, as measured by flow cytometry. (D) Frequency of PD1+ T-effector memory (CD44+CD62L−CCR7lowCD127low) cells in the spleen, as measured by flow cytometry. [file Image_11.JPEG]

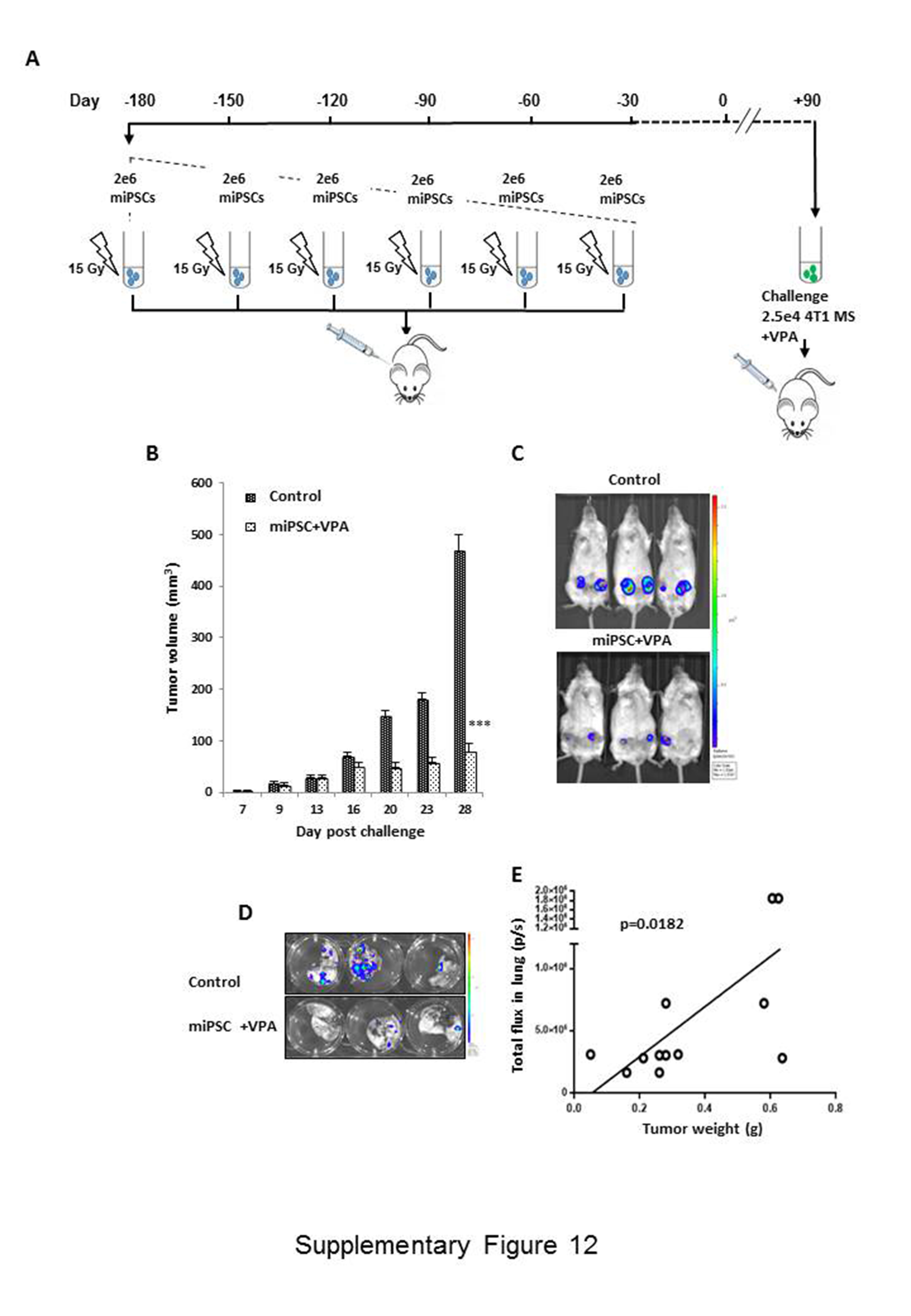

Supplement: Supplementary Figure 12 — Effective memory immune response following vaccination with miPSCs; Mice (n = 6) were treated with 2 × 10^6^ irradiated BALB/c-derived miPSCs during 6 months, with a total of 6 injections, once every 30 days. One hundred twenty days after the last vaccine inoculation, mice were challenged with 2.5 × 10^4^ 4T1Luc cells cultured in mammo sphere condition. Mammo sphere were performed from 4T1 that cultured for 9 days at density of 100,000 cells per well in MEF-conditioned medium (3/4 MEF-conditioned medium + 1/4 mES medium + 4 ng/mL bFGF), and addition of TNF-alpha (20 ng/mL), and TGF-β 1 (10 ng/mL). Mammo spheres were dissociated in PBS/EDTA before injection into both mammary fat-pad glands. Vaccinated mice received 4 mg/mL. VPA by oral route that was started from the day of tumor injection until the sacrifice. Control mice (n = 6) received only PBS. (A) Experimental protocol to evaluate in vivo immune memory generated by vaccination: BALB/c mice were injected subcutaneously six times with 2 × 10^6^ miPSCs (15 Gy irradiated) in the right flank. (B) At day 28 post-challenge, breast tumors were significantly smaller in mice that had undergone the 6-month vaccination protocol compared to unvaccinated mice. (G) Bioluminescence imaging of tumors from mice treated with miPSCs + VPA compared to untreated controls. (H) Bioluminescence imaging of lungs isolated from vaccinated and control mice 28 days after challenge. (I) A significant correlation was found between tumor burden and metastatic spread at day 28. [file Image_12.JPEG]

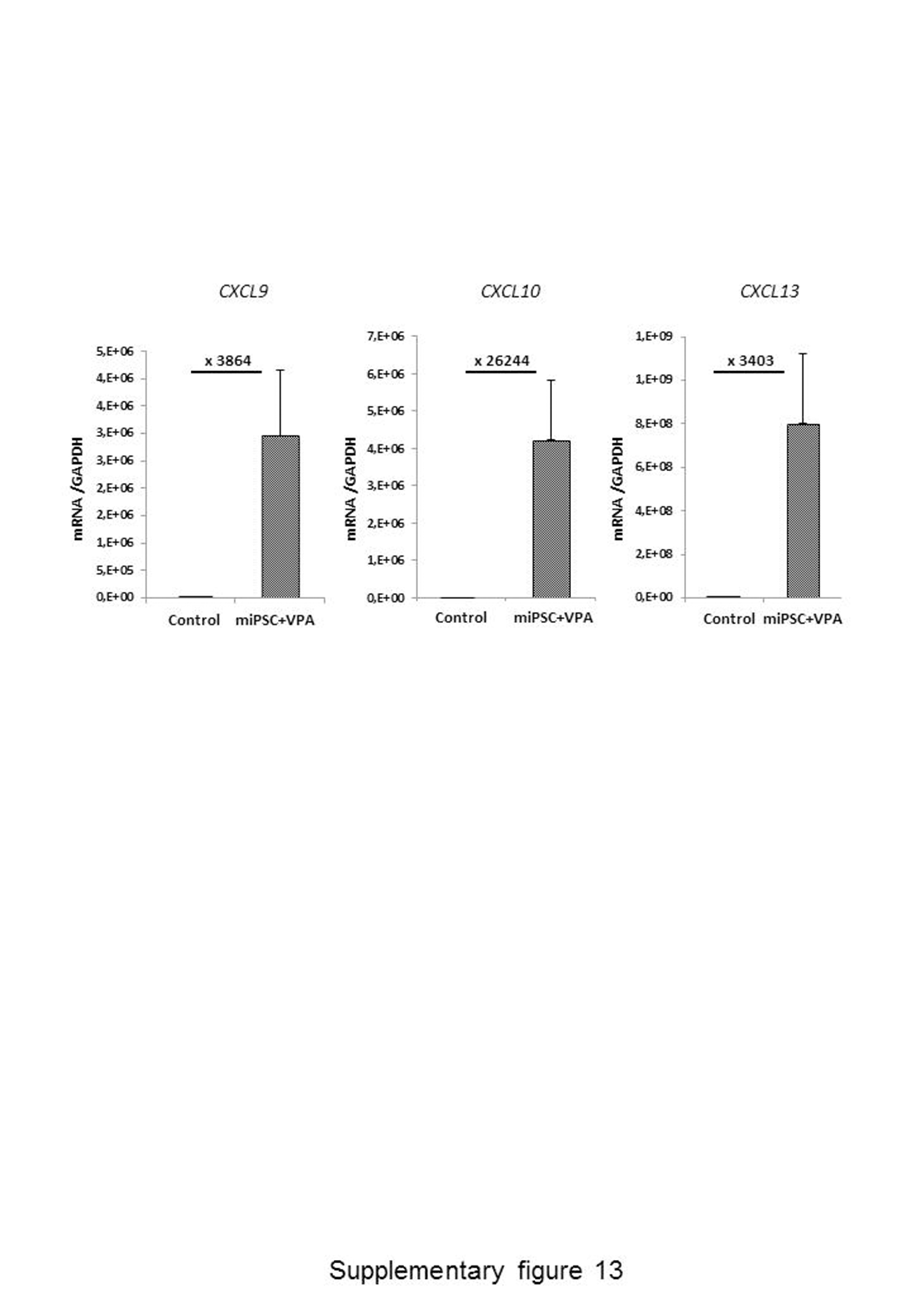

Supplement: Supplementary Figure 13 — CXCL9, CXCL10, and CXCL13 chemokines mRNA expressions in tumors from miPSC + VPA treated (n = 6) and control (n = 6) mice. Mice (n = 6) were treated with sub-cutaneous injections of 2 × 10^6^ irradiated BALB/c-derived miPSCs during 6 months, with a total of 6 injections, once every 30 days. One hundred twenty days after the last vaccine inoculation, mice were challenged with 2.5 × 10^4^ 4T1Luc cells and vaccinated mice received 4 mg/mL VPA by oral route starting from the day of tumor injection until the sacrifice. Control mice (n = 6) received only PBS. After 28 days RNAs of tumors were extracted for the quantification of CXCL9, CXCL10, and CXCL13 mRNA by real time RT-PCR. [file Image_13.JPEG]
