# Supplementary material for: Evidence of Antitumor and Antimetastatic Potential of Induced Pluripotent Stem Cell-Based Vaccines in Cancer Immunotherapy
Source: Front Med (Lausanne). 2021 Dec 10;8:729018. doi: 10.3389/fmed.2021.729018 (PMC8702815; doi:10.3389/fmed.2021.729018)
Supplement: Supplementary Table 1 — List of antibodies; Names, references, and the dilution used. [file Data_Sheet_1.pdf]

Supplementary Table 1

Product name	Product Catalog #	Dilution for one test*
CD44 Antibody, eFluor 450, eBioscience™	48-0441-80	1/80
CD24 Antibody, APC, eBioscience™	17-0242-80	1/300
CD45 Antibody, PE-Cyanine7, eBioscience™	25-0451-81	1/160
CD8a Antibody, APC-eFluor 780, eBioscience™	47-0081-82	1/40
CD25 Antibody, PE, eBioscience™	12-0251-81	1/160
CD279 (PD-1) Antibody, APC, eBioscience™	17-9985-82	1/40
MHC Class I (H-2Kd/H-2Dd), eFluor 450, eBioscience™	48-5998-82	1/20
CD3e Antibody, FITC, eBioscience™	11-0031-82	1/100
CD24 Antibody, VioBlue®, Miltenyi Biotec	130-102-734	1/10
CD11b Antibody,, VioBlue®, Miltenyi Biotec	130-113-800	1/50
Gr-1 Antibody , APC, Miltenyi Biotec	130-102-385	1/10
CD44 Antibody, , APC-Vio® 770, Miltenyi Biotec	130-102-326	1/10
CD4 Antibody, , PE-Vio®770, Miltenyi Biotec	130-124-712	1/50
Ly-6C Antibody, APC-eFluor 780, eBioscience™	47-5932-82	1/40
CXCR5 Antibody , APC, eBioscience™	17-7185-82	1/40
CD22 Antibody, PE-Cyanine 5, Invitrogen	MA5-28653	1/50
CD62L Antibody, FITC , Miltenyi Biotec	130-112-646	1/50
Arginase 1/ARG1 Antibody , PE , R&D	IC5868P	1/10
CD197(CCR7)Antibody, PerCP-Cy™5.5 ,BD Pharmingen™	560812	1/10
CD127 Antiody (A7R34) PE, eBioscience™	1271-81	1/80

**dilution for one test = volume of antibody (µl) staining up to 10^6 cells*

100 µl (buffer volume)
