# Supplementary material for: Evidence of Antitumor and Antimetastatic Potential of Induced Pluripotent Stem Cell-Based Vaccines in Cancer Immunotherapy
Source: Front Med (Lausanne). 2021 Dec 10;8:729018. doi: 10.3389/fmed.2021.729018 (PMC8702815; doi:10.3389/fmed.2021.729018)
Supplement: Supplementary Table 2 — Genes found up regulated in 4T1-transplant samples as compared to 4T1-in vitro done by LIMMA analysis: for each up regulated genes columns present respective statistics obtained by LIMMA algorithm: log2 Fold Change, average expression in microarray, t-statistics, p-value, False discovery rate adjusted p-values and B-statistics. [file Data_Sheet_2.pdf]

Supplementary Table 2 : genes found up regulated in 4T1-transplant samples as compared to 4T1-*in vitro*

gene	logFC	AveExpr	t	P.Value	adj.P.Val	B
H2-Ea-ps	3.15795	7.23913	19.37328	1.60302E-13	4.02394E-10	21.08555
Eno1	2.71495	11.54169	18.72646	2.87708E-13	4.97076E-10	20.53706
Dlx1	2.91217	5.84354	17.63809	8.03301E-13	1.07946E-09	19.56538
Lsm14a	3.07174	8.72571	16.61478	2.22454E-12	2.44578E-09	18.59129
Ppp1r16b	2.24850	4.98073	16.48806	2.53333E-12	2.55317E-09	18.46630
Ccr7	2.80694	5.62976	16.40058	2.77257E-12	2.57934E-09	18.37945
Cpz	2.45889	5.96792	15.75027	5.49715E-12	3.69347E-09	17.71840
Tfec	3.23459	4.98628	15.69894	5.80840E-12	3.69720E-09	17.66505
B3gnt4	2.45081	4.89424	15.26231	9.33895E-12	4.55412E-09	17.20397
Dtd2	2.78285	6.79523	15.13460	1.07544E-11	4.95328E-09	17.06661
Klk1b5	2.50101	3.91951	14.51149	2.17346E-11	8.38664E-09	16.37964
Plcg1	2.87666	7.94580	14.11378	3.45209E-11	1.19164E-08	15.92602
Egfl8	3.06182	6.77746	14.06293	3.66535E-11	1.19164E-08	15.86714
H2-Q5	4.20586	7.45093	14.04491	3.74419E-11	1.19164E-08	15.84624
Rps6kc1	2.47854	6.17354	13.98935	3.99877E-11	1.21905E-08	15.78159
Rasgrp4	2.17516	5.50347	13.97028	4.09028E-11	1.21905E-08	15.75935
Sla2	2.94408	6.56704	13.94132	4.23350E-11	1.21905E-08	15.72551
Gas8	2.56629	7.13002	13.87716	4.56983E-11	1.24193E-08	15.65032
Tnfrsf14	2.55293	6.60905	13.87162	4.60016E-11	1.24193E-08	15.64381
Cdon	2.52881	7.22250	13.84291	4.76081E-11	1.24720E-08	15.61003
Plxna1	2.67608	7.81644	13.59757	6.39893E-11	1.49187E-08	15.31876
Polh	2.89621	6.89254	13.59557	6.41452E-11	1.49187E-08	15.31636
Col9a2	2.91238	5.85019	13.50846	7.13235E-11	1.54033E-08	15.21174
Mlc1	2.05313	4.11019	13.44906	7.66986E-11	1.59930E-08	15.14004
Pip4k2b	3.77113	7.34185	13.42227	7.92606E-11	1.61388E-08	15.10761
Pglyrp1	2.33150	5.18249	13.40058	8.14013E-11	1.61388E-08	15.08130
Gzmg	2.52424	4.11136	13.27812	9.46779E-11	1.73490E-08	14.93205
Farp2	3.05440	7.06315	13.23667	9.96726E-11	1.77271E-08	14.88123
Krtap5-5	2.32828	4.93314	13.10957	1.16786E-10	1.93480E-08	14.72453
3110070M22Rik	2.18151	5.15737	12.90081	1.51918E-10	2.41749E-08	14.46410
Vav1	2.24475	5.29367	12.78302	1.76491E-10	2.73651E-08	14.31548
Hlx	2.08700	6.89985	12.66883	2.04322E-10	2.97719E-08	14.17020
Kirrel	3.81504	7.35539	12.62375	2.16544E-10	3.06935E-08	14.11253
Il10ra	2.27528	5.52758	12.61559	2.18837E-10	3.06935E-08	14.10207
Pik3ap1	2.36424	5.65557	12.59875	2.23652E-10	3.06935E-08	14.08046
a	2.16155	5.60147	12.54340	2.40270E-10	3.15850E-08	14.00928
Mpp4	2.63015	4.72559	12.46989	2.64373E-10	3.36560E-08	13.91429
Srgap1	2.17109	4.66251	12.39020	2.93391E-10	3.58411E-08	13.81075
Scn5a	2.17967	6.53059	12.37643	2.98733E-10	3.61288E-08	13.79281
Atxn1	2.85736	6.56655	12.34188	3.12595E-10	3.70640E-08	13.74770
Maml2	3.09713	6.03512	12.32788	3.18406E-10	3.73022E-08	13.72938

Fbxl15	2.46870	7.21275	12.32224	3.20773E-10	3.73022E-08	13.72201
Prcc	2.98493	7.60643	12.23328	3.60738E-10	4.11581E-08	13.60516
Prf1	2.76297	6.91167	12.05840	4.55298E-10	5.05172E-08	13.37330
Kcnj2	2.53718	3.97130	12.01003	4.85807E-10	5.34123E-08	13.30865
Mall	3.25681	6.61842	11.99170	4.97924E-10	5.42513E-08	13.28409
Mt3	2.16491	5.16258	11.88598	5.74278E-10	6.09238E-08	13.14181
Ccl3	2.54690	5.21462	11.75643	6.84911E-10	6.90702E-08	12.96598
Fam167b	2.44953	5.08227	11.65638	7.85557E-10	7.60042E-08	12.82905
Olfml3	2.10886	6.92265	11.64474	7.98239E-10	7.66182E-08	12.81305
Ikbke	2.83495	6.62363	11.62881	8.15938E-10	7.70934E-08	12.79114
Pglyrp2	2.45045	4.92581	11.59211	8.58302E-10	7.92390E-08	12.74056
Krtap4-2	2.26907	6.40990	11.58444	8.67440E-10	7.94759E-08	12.72997
Lat2	2.35223	5.40001	11.50077	9.74052E-10	8.45308E-08	12.61408
Pou3f3	2.10591	4.32593	11.49235	9.85517E-10	8.45308E-08	12.60238
Zfp865	2.52001	5.99514	11.45876	1.03268E-09	8.79527E-08	12.55563
Mfap4	2.48530	5.48291	11.38362	1.14697E-09	9.56655E-08	12.45062
Dennd1b	2.66653	6.96942	11.34597	1.20914E-09	1.00160E-07	12.39779
Gatm	2.36132	6.76143	11.28359	1.32007E-09	1.07871E-07	12.30993
Tmem59	2.39471	8.65622	11.24330	1.39732E-09	1.13418E-07	12.25297
Spp1	3.23903	11.43275	11.18810	1.51095E-09	1.20388E-07	12.17467
Zdhhc1	2.31291	7.33319	11.18245	1.52312E-09	1.20396E-07	12.16663
Crabp1	2.76487	6.68717	11.17575	1.53769E-09	1.20759E-07	12.15709
Gipc3	2.10537	5.70311	11.13152	1.63752E-09	1.23776E-07	12.09407
Cmklr1	3.16243	5.61387	11.12125	1.66167E-09	1.24821E-07	12.07940
Chmp4c	3.29197	6.09680	11.08776	1.74302E-09	1.28537E-07	12.03149
Ankrd27	2.19429	7.52373	11.08062	1.76090E-09	1.29069E-07	12.02126
Spock3	2.37772	3.35600	11.05980	1.81412E-09	1.29557E-07	11.99141
Usp42	2.21663	7.64128	11.00710	1.95658E-09	1.36779E-07	11.91562
Pdk3	2.70347	7.65561	10.94900	2.12726E-09	1.44663E-07	11.83174
Bard1	2.39200	6.93920	10.94731	2.13244E-09	1.44663E-07	11.82929
Tnnt2	3.50343	8.78599	10.82187	2.55748E-09	1.63652E-07	11.64691
Thap2	2.32926	6.35892	10.81689	2.57608E-09	1.63975E-07	11.63964
Igsf6	2.36439	5.58132	10.80849	2.60777E-09	1.64853E-07	11.62736
Cdr2l	2.06543	6.25393	10.78013	2.71786E-09	1.70310E-07	11.58585
Mybl1	3.11526	5.96010	10.77377	2.74321E-09	1.71012E-07	11.57653
Fgf8	2.43848	4.71359	10.76575	2.77552E-09	1.72139E-07	11.56477
Cit	2.31206	7.19638	10.70829	3.01906E-09	1.84280E-07	11.48031
Stra6	3.74145	6.94526	10.66220	3.23057E-09	1.92465E-07	11.41229
Col12a1	3.03910	6.88634	10.61986	3.43854E-09	2.02465E-07	11.34961
Fhl3	2.74349	7.74697	10.60217	3.52954E-09	2.05222E-07	11.32336
Ammecr1	2.71633	7.16867	10.59542	3.56491E-09	2.06287E-07	11.31334
Lypd3	2.61587	7.06339	10.56570	3.72524E-09	2.14538E-07	11.26912
Tssk6	2.04415	5.56189	10.55282	3.79698E-09	2.16791E-07	11.24995
Papln	2.46176	5.47610	10.52655	3.94793E-09	2.22076E-07	11.21076
Cdx1	2.26472	5.11947	10.40951	4.70095E-09	2.48267E-07	11.03521

Irg1	2.66979	5.45868	10.35825	5.07672E-09	2.65791E-07	10.95784
C1qtnf3	2.15985	4.90882	10.34042	5.21468E-09	2.69514E-07	10.93086
Mcpt1	2.15805	4.53349	10.33084	5.29041E-09	2.71436E-07	10.91635
Bcl7a	2.24739	7.16311	10.30973	5.46148E-09	2.76063E-07	10.88433
Itk	2.07315	4.59638	10.30218	5.52400E-09	2.76063E-07	10.87287
Pcdhb15	2.28633	4.72930	10.26855	5.81187E-09	2.84770E-07	10.82174
Rnf219	2.73773	6.48236	10.21357	6.31688E-09	3.04368E-07	10.73786
Itga2	2.23428	9.26892	10.19322	6.51530E-09	3.10220E-07	10.70672
Stk11ip	2.11958	6.17146	10.15820	6.87211E-09	3.22753E-07	10.65302
Ppard	3.62903	7.92393	10.09785	7.53573E-09	3.44468E-07	10.56017
Ptk7	2.42804	7.52839	10.09572	7.56037E-09	3.44468E-07	10.55688
Serpinb1a	2.07561	5.03589	10.07857	7.76171E-09	3.49086E-07	10.53040
P2ry2	2.75998	6.17790	10.05789	8.01183E-09	3.54927E-07	10.49845
Cdc27	2.14309	9.54029	10.03242	8.33169E-09	3.65085E-07	10.45900
Slc25a24	2.02126	8.73484	9.99379	8.84237E-09	3.80509E-07	10.39905
Akt3	2.67527	8.57326	9.98158	9.01048E-09	3.82584E-07	10.38007
Mad1l1	2.20222	7.23014	9.96172	9.29125E-09	3.87477E-07	10.34914
Zfp369	2.59562	6.66613	9.90548	1.01369E-08	4.07514E-07	10.26133
Itga4	2.06227	5.68196	9.89610	1.02856E-08	4.10541E-07	10.24664
Kif21b	2.75905	6.23320	9.87380	1.06483E-08	4.22233E-07	10.21169
Brinp1	2.08050	3.70576	9.86499	1.07952E-08	4.25269E-07	10.19787
Cpa1	2.00733	5.22213	9.85370	1.09868E-08	4.30015E-07	10.18013
Dpysl2	2.61013	9.36641	9.83257	1.13547E-08	4.38736E-07	10.14691
Gpa33	3.07475	9.07092	9.80285	1.18941E-08	4.53777E-07	10.10010
Ifi44l	2.17412	5.11704	9.77301	1.24629E-08	4.72497E-07	10.05298
Abhd2	2.72951	9.33211	9.72633	1.34100E-08	5.03667E-07	9.97908
Pask	2.34486	6.18843	9.70510	1.38657E-08	5.14390E-07	9.94537
Ly9	2.78092	5.48761	9.66848	1.46897E-08	5.33123E-07	9.88710
Gzmc	2.11817	4.44670	9.62007	1.58583E-08	5.65323E-07	9.80983
Pla2g5	2.35270	4.96407	9.61609	1.59587E-08	5.65323E-07	9.80347
Cep290	2.22880	6.30803	9.58084	1.68765E-08	5.93326E-07	9.74702
Nuf2	2.33474	7.97119	9.56448	1.73211E-08	6.03692E-07	9.72076
Chtf18	2.49171	6.41142	9.50163	1.91464E-08	6.54114E-07	9.61959
6820431F20Rik	2.06532	10.46377	9.49318	1.94066E-08	6.61135E-07	9.60595
Cacnb2	2.20476	4.34962	9.48860	1.95494E-08	6.62955E-07	9.59855
Dusp9	2.99660	7.12695	9.47766	1.98944E-08	6.68381E-07	9.58088
Ucn	2.18053	5.64106	9.47763	1.98956E-08	6.68381E-07	9.58082
Gcnt1	2.57143	5.48484	9.46992	2.01424E-08	6.72934E-07	9.56836
Igfbp3	2.01025	7.03139	9.46812	2.02006E-08	6.73019E-07	9.56545
Plk3	2.64140	6.94037	9.40301	2.24263E-08	7.13746E-07	9.45986
Prr5	2.36473	6.41431	9.39964	2.25483E-08	7.15699E-07	9.45438
Myo1f	2.11304	6.03681	9.38917	2.29315E-08	7.20347E-07	9.43735
Zscan2	2.76856	6.73176	9.35730	2.41411E-08	7.48622E-07	9.38541
Snx22	2.44466	7.12393	9.34416	2.46589E-08	7.60779E-07	9.36397
Vav2	2.15463	6.46061	9.33394	2.50695E-08	7.69519E-07	9.34728

Cdc25c	2.52477	6.80544	9.32929	2.52588E-08	7.71862E-07	9.33967
Sp5	2.01228	4.54878	9.30992	2.60633E-08	7.88023E-07	9.30799
Ccl7	2.33499	5.88682	9.28940	2.69452E-08	8.02647E-07	9.27436
Mki67	2.55250	9.99102	9.23289	2.95376E-08	8.62870E-07	9.18150
Onecut3	2.09295	4.65010	9.22000	3.01651E-08	8.76963E-07	9.16025
Gng2	2.51666	7.30569	9.13149	3.48647E-08	9.82875E-07	9.01384
Lats1	2.26979	8.72874	9.11206	3.59952E-08	1.00305E-06	8.98157
Sgol1	2.37313	7.75504	9.09769	3.68557E-08	1.01073E-06	8.95767
Cd276	2.40148	8.59387	9.09380	3.70924E-08	1.01346E-06	8.95119
Cdk6	2.25001	8.36690	9.09330	3.71227E-08	1.01346E-06	8.95037
C330027C09Rik	2.53602	8.14717	9.08537	3.76108E-08	1.01988E-06	8.93716
Tram2	2.24814	7.97903	9.08334	3.77366E-08	1.02100E-06	8.93378
Stk4	2.94934	6.64982	9.05068	3.98245E-08	1.06793E-06	8.87931
Peg12	2.88215	4.59727	9.02715	4.14029E-08	1.09715E-06	8.83999
Gzmd	2.89939	4.89842	9.01157	4.24845E-08	1.11455E-06	8.81389
Spry4	2.12256	6.51694	9.00900	4.26657E-08	1.11688E-06	8.80959
Abr	2.41979	8.04580	8.97829	4.48942E-08	1.15765E-06	8.75808
Dclre1b	2.27210	7.17737	8.97685	4.50011E-08	1.15765E-06	8.75567
Ubxn2b	2.02020	7.01927	8.97446	4.51805E-08	1.15765E-06	8.75164
Runx1	2.41027	8.93371	8.95033	4.70286E-08	1.18826E-06	8.71107
Xylt2	2.55010	6.54213	8.94989	4.70627E-08	1.18826E-06	8.71034
Tnfrsf10b	2.46582	7.77538	8.92324	4.91978E-08	1.22487E-06	8.66544
Il18bp	2.04442	6.31734	8.92294	4.92218E-08	1.22487E-06	8.66495
Mastl	2.09357	7.87193	8.89978	5.11606E-08	1.26243E-06	8.62585
Map3k11	2.11926	6.65765	8.88915	5.20763E-08	1.27914E-06	8.60789
Zmiz1	2.32331	9.03616	8.87276	5.35226E-08	1.31033E-06	8.58017
B4galt5	2.37024	8.53122	8.87008	5.37634E-08	1.31356E-06	8.57562
Arhgap10	2.23864	7.73741	8.85793	5.48675E-08	1.33515E-06	8.55505
Ifi202b	3.13614	7.62310	8.84986	5.56140E-08	1.34783E-06	8.54137
Ltb	2.10527	5.52030	8.81332	5.91303E-08	1.42171E-06	8.47931
Pdzd9	2.30749	5.95028	8.79510	6.09689E-08	1.44864E-06	8.44830
Cdca7	2.02539	7.35098	8.79269	6.12168E-08	1.45168E-06	8.44420
Cyp4f18	2.08286	5.22437	8.77154	6.34349E-08	1.48679E-06	8.40816
Cap1	2.64541	10.24332	8.75471	6.52608E-08	1.51574E-06	8.37943
Abhd8	2.37312	6.05049	8.73859	6.70609E-08	1.54483E-06	8.35188
Inhba	3.27559	8.65235	8.73602	6.73528E-08	1.54566E-06	8.34748
Gsg2	2.04481	5.92256	8.71855	6.93719E-08	1.58480E-06	8.31757
Panx1	2.07352	6.06395	8.71676	6.95823E-08	1.58480E-06	8.31450
Plscr1	2.16335	7.90286	8.71465	6.98305E-08	1.58746E-06	8.31090
Sema3e	2.47766	7.02370	8.65679	7.70286E-08	1.71879E-06	8.21154
Rad51	2.19403	7.56776	8.65439	7.73428E-08	1.72249E-06	8.20742
Traf1	2.36788	6.71496	8.61781	8.23102E-08	1.80337E-06	8.14437
Ccdc130	2.39144	6.81455	8.59492	8.55870E-08	1.85108E-06	8.10482
Dvl3	2.71968	7.50523	8.56245	9.04706E-08	1.92563E-06	8.04861
Cenpe	2.40716	8.62523	8.56193	9.05514E-08	1.92563E-06	8.04770

Hcls1	2.20596	5.75510	8.55771	9.12069E-08	1.93180E-06	8.04040
Esm1	2.03478	4.39375	8.54702	9.28912E-08	1.95719E-06	8.02186
Nt5e	2.65748	9.53693	8.54105	9.38465E-08	1.97045E-06	8.01149
Ttll3	2.03263	6.98013	8.53147	9.54014E-08	1.99617E-06	7.99484
Itgb3	3.17481	7.33314	8.51869	9.75161E-08	2.02988E-06	7.97263
Grasp	2.44079	7.01752	8.49985	1.00723E-07	2.07521E-06	7.93984
Zfp948	2.11119	7.17311	8.44482	1.10736E-07	2.22836E-06	7.84381
Ptgs2	2.37211	9.50539	8.43773	1.12100E-07	2.24832E-06	7.83140
Ocstamp	2.18618	5.20451	8.42929	1.13746E-07	2.26256E-06	7.81663
Ccnf	2.14057	8.21471	8.42294	1.15000E-07	2.28002E-06	7.80552
Sema3f	3.00923	8.37909	8.41902	1.15782E-07	2.28070E-06	7.79865
Maf	2.54129	5.76423	8.41334	1.16924E-07	2.29559E-06	7.78870
Slc6a1	2.03027	3.99897	8.39486	1.20725E-07	2.35113E-06	7.75628
Gzmf	2.02137	4.68490	8.37949	1.23985E-07	2.39980E-06	7.72927
Top2a	2.03296	9.96171	8.35119	1.30227E-07	2.48809E-06	7.67949
Bmpr2	2.20846	9.46484	8.32948	1.35238E-07	2.56358E-06	7.64122
Taf1d	2.04141	9.86326	8.31315	1.39139E-07	2.60124E-06	7.61239
Ntng2	2.35706	5.86696	8.30917	1.40108E-07	2.61067E-06	7.60536
Med14	2.05066	9.50285	8.28409	1.46373E-07	2.69033E-06	7.56101
Mmgt2	2.36088	7.08310	8.26801	1.50545E-07	2.74614E-06	7.53252
Tnfrsf11b	2.33025	5.04728	8.25977	1.52729E-07	2.77343E-06	7.51792
2900097C17Rik	2.36708	7.92681	8.25496	1.54020E-07	2.77737E-06	7.50938
Zfp626	2.01296	5.98106	8.24756	1.56030E-07	2.79559E-06	7.49624
Peak1	2.39922	9.35839	8.23383	1.59828E-07	2.85097E-06	7.47186
Gcc2	2.15694	7.41422	8.19537	1.70987E-07	2.99699E-06	7.40343
Timp1	2.43578	9.63522	8.18443	1.74310E-07	3.02453E-06	7.38391
Ikzf2	3.06770	6.34190	8.07164	2.12737E-07	3.46528E-06	7.18188
Smad3	2.01808	9.31621	8.05346	2.19712E-07	3.53821E-06	7.14916
Ect2	2.89002	8.91995	8.04284	2.23900E-07	3.57781E-06	7.13001
Trpm8	2.07306	3.53872	8.03020	2.28988E-07	3.64873E-06	7.10722
Bcl11b	2.32190	4.68255	7.95905	2.59975E-07	3.99509E-06	6.97849
Bub1	2.27309	8.71692	7.94645	2.65902E-07	4.06038E-06	6.95562
Insl6	2.70892	7.10914	7.94402	2.67059E-07	4.07291E-06	6.95121
Trpv4	2.28558	7.18223	7.89612	2.91018E-07	4.33147E-06	6.86406
Cpne2	2.12733	7.67930	7.89513	2.91534E-07	4.33147E-06	6.86226
Ankrd1	2.36669	8.79218	7.89380	2.92235E-07	4.33655E-06	6.85982
Thbs1	2.38818	9.96105	7.88096	2.99059E-07	4.38402E-06	6.83641
Map1b	2.17104	9.10630	7.87472	3.02434E-07	4.42813E-06	6.82502
Cabp7	2.08521	4.57308	7.87174	3.04063E-07	4.44660E-06	6.81957
Plau	2.27504	8.26796	7.84130	3.21206E-07	4.63168E-06	6.76392
S100g	2.19944	4.47292	7.84081	3.21488E-07	4.63168E-06	6.76303
Tubb6	2.11183	8.97447	7.81999	3.33800E-07	4.73824E-06	6.72490
Rrp9	2.04193	7.61429	7.79643	3.48329E-07	4.88711E-06	6.68167
Mgat5	2.64212	7.75553	7.78065	3.58429E-07	4.97468E-06	6.65267
E2f8	2.66939	8.35046	7.78024	3.58695E-07	4.97468E-06	6.65192

Klrc1	2.20457	3.63330	7.74434	3.82835E-07	5.19997E-06	6.58583
Vdr	2.19786	8.06625	7.74346	3.83445E-07	5.19997E-06	6.58422
Olig2	2.69647	4.98194	7.72855	3.93980E-07	5.32225E-06	6.55671
Melk	2.94603	7.22853	7.70072	4.14456E-07	5.50486E-06	6.50530
Ccnb2	3.11667	9.59590	7.69743	4.16949E-07	5.52310E-06	6.49921
Krt34	2.08102	4.55096	7.66525	4.42176E-07	5.76258E-06	6.43960
Pstpip1	2.02293	7.29944	7.63278	4.69232E-07	6.04355E-06	6.37933
Shcbp1	2.06243	7.76294	7.63170	4.70166E-07	6.04913E-06	6.37731
Lrp5	2.34548	8.30598	7.62989	4.71727E-07	6.06277E-06	6.37395
Nt5c3b	2.22207	8.85951	7.60112	4.97278E-07	6.29088E-06	6.32042
Moxd1	2.50388	8.54332	7.56445	5.31963E-07	6.64624E-06	6.25199
Mmp10	2.51634	7.20806	7.51999	5.77404E-07	7.06405E-06	6.16880
Sp100	2.50528	7.21474	7.51448	5.83312E-07	7.12583E-06	6.15846
2610020H08Rik	2.14646	5.67528	7.50119	5.97810E-07	7.25894E-06	6.13355
Lrrc49	2.26309	6.50280	7.50041	5.98675E-07	7.26216E-06	6.13208
Nemf	2.32066	8.45222	7.48621	6.14619E-07	7.38886E-06	6.10540
Proser2	2.53822	6.45675	7.42732	6.85567E-07	8.04976E-06	5.99452
Mmp9	2.37562	9.62117	7.41702	6.98829E-07	8.12657E-06	5.97508
Gm20767	2.04638	4.18407	7.38081	7.47592E-07	8.57816E-06	5.90661
Ccdc82	2.03710	7.17215	7.35516	7.84259E-07	8.87442E-06	5.85801
Zfp81	2.62392	6.35922	7.34514	7.99101E-07	8.98172E-06	5.83898
Ldb1	2.06550	9.80043	7.29924	8.70846E-07	9.56586E-06	5.75171
Efna5	3.24979	7.58518	7.28989	8.86270E-07	9.66583E-06	5.73389
Dusp7	2.01257	9.02781	7.28984	8.86341E-07	9.66583E-06	5.73381
Apbb1ip	2.29681	7.63706	7.28089	9.01365E-07	9.78555E-06	5.71674
Padi4	2.79924	7.88583	7.27947	9.03773E-07	9.80290E-06	5.71403
Slc41a2	2.02530	6.52458	7.26035	9.36868E-07	1.00895E-05	5.67753
Kif23	2.25099	8.82194	7.22166	1.00771E-06	1.05976E-05	5.60354
Sfn	3.45550	7.41093	7.17550	1.09957E-06	1.12127E-05	5.51498
Atp10a	2.83231	6.54746	7.17021	1.11064E-06	1.13065E-05	5.50481
H2-Q2	2.36756	5.85351	7.14015	1.17579E-06	1.18106E-05	5.44695
Hal	2.04654	6.00803	7.13271	1.19252E-06	1.19489E-05	5.43261
Slfn3	2.35754	6.45777	7.07162	1.33953E-06	1.29706E-05	5.31461
Etv4	2.01407	7.54970	7.05806	1.37463E-06	1.31577E-05	5.28835
Depdc1a	2.97552	7.17988	7.02144	1.47432E-06	1.38651E-05	5.21728
Has2	2.51342	8.62707	7.01862	1.48231E-06	1.39293E-05	5.21180
Ube2l6	2.84963	6.65969	7.01350	1.49691E-06	1.39889E-05	5.20186
Tslp	2.64231	8.37438	6.99055	1.56421E-06	1.44232E-05	5.15721
G6pd2	2.04258	5.69904	6.99039	1.56468E-06	1.44232E-05	5.15691
Rin1	2.02939	7.41593	6.96808	1.63316E-06	1.49180E-05	5.11343
Ccnb1	2.07663	8.53211	6.96464	1.64398E-06	1.49942E-05	5.10673
Phlda2	2.84794	8.19156	6.96404	1.64590E-06	1.49997E-05	5.10554
Tmem97	2.46255	8.68307	6.96367	1.64707E-06	1.49997E-05	5.10482
Ror2	2.15439	5.71353	6.95698	1.66836E-06	1.51673E-05	5.09178
Bst1	2.35734	6.84276	6.94815	1.69697E-06	1.53272E-05	5.07452

Il12rb1	2.07429	6.07336	6.94520	1.70662E-06	1.53582E-05	5.06877
Tnfsf10	2.12462	5.49378	6.90073	1.85934E-06	1.62712E-05	4.98178
Upp1	2.37070	7.73967	6.89586	1.87692E-06	1.63681E-05	4.97222
Msln	3.21157	7.48685	6.84793	2.05923E-06	1.75541E-05	4.87814
Il23a	2.42213	7.86287	6.79062	2.30161E-06	1.92235E-05	4.76521
Kcnn4	2.01903	8.42918	6.78198	2.34062E-06	1.94553E-05	4.74815
Nos2	3.38926	6.44790	6.77025	2.39470E-06	1.97825E-05	4.72496
Mxd1	2.35196	7.29222	6.76770	2.40662E-06	1.98673E-05	4.71993
Tnc	2.78273	8.69423	6.74698	2.50587E-06	2.05186E-05	4.67891
Ngfr	2.07643	6.93356	6.73061	2.58726E-06	2.09580E-05	4.64648
Pthlh	2.41838	6.48930	6.69839	2.75552E-06	2.19534E-05	4.58254
Oasl2	2.19736	8.19181	6.68539	2.82661E-06	2.23518E-05	4.55669
P2ry6	2.56314	7.10792	6.66690	2.93097E-06	2.30926E-05	4.51990
Scx	2.21106	6.83030	6.66295	2.95375E-06	2.31659E-05	4.51204
Slc15a3	2.89646	6.81801	6.65259	3.01446E-06	2.35509E-05	4.49140
Snapc5	2.01164	6.36566	6.61684	3.23399E-06	2.47857E-05	4.42007
Tcfl5	2.03529	5.51727	6.53774	3.78063E-06	2.80681E-05	4.26162
B4galnt1	2.00030	8.21844	6.49602	4.10658E-06	2.99004E-05	4.17772
Rara	2.76441	7.68741	6.48699	4.18088E-06	3.03321E-05	4.15953
Slc7a5	2.05166	9.22727	6.42705	4.71069E-06	3.31420E-05	4.03850
Hhip	2.45632	7.83886	6.42547	4.72558E-06	3.31696E-05	4.03530
Plek2	2.46206	7.36816	6.40842	4.88913E-06	3.39628E-05	4.00079
Hsf2	2.37737	6.22928	6.40749	4.89824E-06	3.39870E-05	3.99890
Zmynd19	2.56685	7.59453	6.40499	4.92272E-06	3.41372E-05	3.99385
Mmp12	3.36345	6.75099	6.38676	5.10541E-06	3.50226E-05	3.95689
Vegfc	2.11973	6.79740	6.37584	5.21815E-06	3.54110E-05	3.93474
Arhgap29	2.16443	7.62573	6.36769	5.30395E-06	3.58025E-05	3.91820
Il1a	3.60965	7.61758	6.32793	5.74415E-06	3.80947E-05	3.83734
Osr2	2.23103	5.98767	6.32127	5.82145E-06	3.84015E-05	3.82378
Itga5	2.05188	7.70941	6.30971	5.95832E-06	3.91205E-05	3.80022
Pole	2.21932	7.40300	6.30898	5.96706E-06	3.91520E-05	3.79873
Ly75	2.78977	7.07523	6.29288	6.16343E-06	4.01187E-05	3.76590
Ube2c	2.09250	9.57980	6.26736	6.48844E-06	4.16517E-05	3.71379
Cxcl11	2.15140	5.99655	6.25584	6.64101E-06	4.24281E-05	3.69023
Sh3bp1	2.05219	7.78584	6.23142	6.97667E-06	4.40476E-05	3.64024
Chek1	2.13426	8.65385	6.23002	6.99641E-06	4.40892E-05	3.63737
BC147527	2.23707	4.04617	6.22743	7.03305E-06	4.42318E-05	3.63208
Gbx2	2.05758	4.83172	6.21805	7.16777E-06	4.48226E-05	3.61284
Pla2g7	2.62805	8.44068	6.19343	7.53396E-06	4.64639E-05	3.56233
Pgf	2.23946	7.26407	6.19200	7.55585E-06	4.65041E-05	3.55939
Kmt2d	2.11017	7.48596	6.13285	8.51956E-06	5.06393E-05	3.43771
Ibsp	2.11184	6.15128	6.10747	8.97116E-06	5.26442E-05	3.38537
Mid1	2.72343	6.14571	6.04417	1.02084E-05	5.81078E-05	3.25445
Glipr2	2.07042	7.62079	5.98967	1.14142E-05	6.30046E-05	3.14134
Id2	2.14463	9.22902	5.90335	1.36327E-05	7.22814E-05	2.96145

Tnrc18	2.58063	7.28845	5.84830	1.52754E-05	7.90235E-05	2.84624
Cd109	2.26403	6.33159	5.74681	1.88592E-05	9.32472E-05	2.63292
Aim2	2.43551	4.63477	5.72860	1.95887E-05	9.57556E-05	2.59451
Ctsw	2.21271	6.87105	5.67561	2.18807E-05	1.03897E-04	2.48256
Mmp13	4.49162	7.64761	5.67297	2.20019E-05	1.04350E-04	2.47697
Wisp1	2.23721	6.51477	5.63358	2.38938E-05	1.11100E-04	2.39354
Tbc1d8b	2.58410	6.70728	5.62693	2.42294E-05	1.12272E-04	2.37943
Zbp1	2.08580	6.72420	5.56340	2.76902E-05	1.24400E-04	2.24443
H2-T24	2.27723	6.21542	5.44665	3.54336E-05	1.51319E-04	1.99523
Peg10	2.16409	5.13338	5.43318	3.64599E-05	1.54555E-04	1.96639
Espn	2.46976	6.25760	5.42585	3.70315E-05	1.56321E-04	1.95067
Trpm6	2.32912	6.73945	5.22646	5.66550E-05	2.18108E-04	1.52146
Gjb3	2.56356	8.11727	5.11422	7.21142E-05	2.62458E-04	1.27820
Cxcl10	2.30243	6.57943	5.05754	8.14974E-05	2.88787E-04	1.15496
