# Supplementary material for: Evidence of Antitumor and Antimetastatic Potential of Induced Pluripotent Stem Cell-Based Vaccines in Cancer Immunotherapy
Source: Front Med (Lausanne). 2021 Dec 10;8:729018. doi: 10.3389/fmed.2021.729018 (PMC8702815; doi:10.3389/fmed.2021.729018)
Supplement: Supplementary Table 3 — Differentially expressed immune genes that were upregulated in 4T1 cells by treatment with valproic acid. The list highlights immune-associated genes that were found to be significantly overexpressed in the treatment group using the Significance Analysis of Microarray algorithm with a false discovery rate of <5%. Columns include: gene symbol, gene description, gene ID number, and the fold-change in expression between the VPA-treated group and untreated controls. [file Data_Sheet_3.pdf]

Supplemental Table 3 : Differentially expressed immune genes that were upregulated in 4T1 cells by treatment with valproic acid

Gene symbol	Description	Gene ID	Fold change (Valproic acid /control)
CD74	CD74 molecule	972	2.756137
TNFRSF9	TNF receptor superfamily member 9	3604	2.152303
CCL2	C-C motif chemokine ligand 2	6347	2.0778043
CASP4	caspase 4	837	1.812713
AREG	amphiregulin	374	1.7866501
IFIT2	interferon induced protein with tetratricopeptide repeats 2	3433	1.7681799
SERPINB2	serpin family B member 2	5055	1.7294099
LY6E	lymphocyte antigen 6 family member E	4061	1.7189492
PLK2	polo like kinase 2	10769	1.6721709
EGR1	early growth response 1	1958	1.6136316
DRAM1	DNA damage regulated autophagy modulator 1	55332	1.6093787
KLF9	Kruppel like factor 9	687	1.6086748
IFIH1	interferon induced with helicase C domain 1	64135	1.5915328
UBE2L6	ubiquitin conjugating enzyme E2 L6	9246	1.559597
IL15RA	interleukin 15 receptor subunit alpha	3601	1.5306206
CCRL2	C-C motif chemokine receptor like 2	9034	1.524512
STAT1	signal transducer and activator of transcription 1	6772	1.4973801
NLRC5	NLR family CARD domain containing 5	84166	1.4913481
ISOC1	isochorismatase domain containing 1	51015	1.4899585
BST2	bone marrow stromal cell antigen 2	684	1.4610225
IL15	interleukin 15	3600	1.4604695
SAMD9L	sterile alpha motif domain containing 9 like	219285	1.448133
HERC6	HECT and RLD domain containing E3 ubiquitin protein ligase family member 6	55008	1.4128106
DHX58	DExH-box helicase 58	79132	1.4095724
CSF1	colony stimulating factor 1	1435	1.401427
SOD2	superoxide dismutase 2	6648	1.3938667
PSMB9	proteasome subunit beta 9	5698	1.3776621
SLC2A6	solute carrier family 2 member 6	11182	1.3619148
RNF213	ring finger protein 213	57674	1.3537916
IL7	interleukin 7	3574	1.3391039
TUBB2A	tubulin beta 2A class IIa	7280	1.331755
TDRD7	tudor domain containing 7	23424	1.3295585
ATP2B1	ATPase plasma membrane Ca2+ transporting 1	490	1.3081365
CCL20	C-C motif chemokine ligand 20	6364	1.307062
CD274	CD274 molecule	29126	1.2911943
CEBPD	CCAAT/enhancer binding protein delta	1052	1.2831194
ZBTB10	zinc finger and BTB domain containing 10	65986	1.2745908
IRS2	insulin receptor substrate 2	8660	1.2700346
NCOA7	nuclear receptor coactivator 7	135112	1.2286383
REL	REL proto-oncogene, NF-kB subunit	5966	1.2227736
CCL7	C-C motif chemokine ligand 7	6354	1.1728142
IL10RA	interleukin 10 receptor subunit alpha	3587	1.1703566
SP110	SP110 nuclear body protein	3431	1.1379845
MSC	musculin	9242	1.1052907
