# Supplementary material for: Evidence of Antitumor and Antimetastatic Potential of Induced Pluripotent Stem Cell-Based Vaccines in Cancer Immunotherapy
Source: Front Med (Lausanne). 2021 Dec 10;8:729018. doi: 10.3389/fmed.2021.729018 (PMC8702815; doi:10.3389/fmed.2021.729018)
Supplement: Supplementary Table 4 — Genes that were differentially expressed between vaccinated and control mice that developed 4T1-derived tumors. Differentially expressed genes are listed with their gene symbol, description, the fold-change in expression found in transcriptome experiments (Vaccine/Control), and the p-value of the comparison. [file Data_Sheet_4.pdf]

Supplementary Table 4 : Genes that were differentially expressed between vaccinated and control mice that developed 4T1-derived tumors.

Gene symbol	Description	FC vaccin/CT 4T1	Ranking p-values
Cxcl13	chemokine (C-X-C motif) ligand 13	8,877	5.1787803E-5
Gm17482	predicted gene, 17482 [Source:MGI Symbol;Acc:MGI:4937116]	3,117	1.3509862E-6
Mir675	microRNA 675; H19, imprinted maternally expressed transcript	2,667	1.8913806E-5
Clec4a1	C-type lectin domain family 4, member a1	2,235	1.6932361E-4
Fxyd1	FXYD domain-containing ion transport regulator 1	2,042	2.6119065E-5
Il20rb	interleukin 20 receptor beta	2,014	3.3324326E-5
Sh2d4a	SH2 domain containing 4A	1,945	5.439971E-4
Egln3	EGL nine homolog 3	1,939	4.6158695E-4
Serpinb2	serine (or cysteine) peptidase inhibitor, clade B, member 2	1,886	3.449518E-4
Clec12a	C-type lectin domain family 12, member a	1,840	0.0011415834
Lmo7	LIM domain only 7	1,828	1.6707196E-4
Vps33b	vacuolar protein sorting 33B (yeast)	1,828	3.62965E-4
Pmp22	peripheral myelin protein 22	1,815	1.1618481E-4
Ghr	growth hormone receptor	1,809	7.3583715E-4
Ly96	lymphocyte antigen 96	1,759	7.488967E-4
Cldn9	claudin 9	1,747	2.193101E-4
9230104L09Rik	RIKEN cDNA 9230104L09 gene	1,741	2.3011799E-4
Ccl22	chemokine (C-C motif) ligand 22	1,729	4.0664684E-4
Ttll1	tubulin tyrosine ligase-like 1	1,717	8.475187E-4
Naf1	nuclear assembly factor 1 homolog	1,711	6.4351974E-4
Enpp2	ectonucleotide pyrophosphatase/phosphodiesterase 2	1,699	6.5387733E-4
Tc2n	tandem C2 domains, nuclear	1,699	0.0012348014
Slc6a8	solute carrier family 6 (neurotransmitter transporter, creatine), r	1,693	8.2500227E-4
Shroom3	shroom family member 3	1,688	8.290552E-4
Zfp595	zinc finger protein 595	1,688	6.4351974E-4
Sod3	superoxide dismutase 3, extracellular	1,676	4.3952087E-4
Cd46	CD46 antigen, complement regulatory protein	1,670	6.642349E-4
Cd72	CD72 antigen	1,670	0.0017567324
Pid1	phosphotyrosine interaction domain containing 1	1,670	0.0011983248
Psmg1	proteasome (prosome, macropain) assembly chaperone 1	1,664	0.0011478879
Rnf223	ring finger 223	1,664	6.421688E-4
Cd300e	CD300e antigen	1,641	0.0011577952
Csf2ra	colony stimulating factor 2 receptor, alpha	1,641	0.002091777
Hnmt	histamine N-methyltransferase	1,641	0.0011888678
Cpa3	carboxypeptidase A3, mast cell	1,630	0.0023011798
Olfml2b	olfactomedin-like 2B	1,625	0.0013100064
Rassf9	Ras association (RalGDS/AF-6) domain family (N-terminal) memb	1,625	0.001398721
Bin2	bridging integrator 2	1,619	0.0028267135
Ms4a14	PREDICTED: membrane-spanning 4-domains, subfamily A, mem	1,619	0.0020715122
Olfr384	olfactory receptor 384	1,613	7.7321444E-4
Hormad2	HORMA domain containing 2	1,608	0.0018985859
Gfra2	glial cell line derived neurotrophic factor family receptor alpha 2	1,597	0.0012388544
Blk	B lymphoid kinase	1,586	9.051608E-4
Ms4a7	membrane-spanning 4-domains, subfamily A, member 7	1,569	0.0021237503
Smpd4	sphingomyelin phosphodiesterase 4	1,564	0.0023291004
Ccr2	chemokine (C-C motif) receptor 2	1,553	0.0021359092
Hhip	Hedgehog-interacting protein	1,553	0.0018076196
Ctse	cathepsin E	1,548	0.0041119517
Gm2573	PREDICTED: predicted gene 2573 (Gm2573)	1,542	0.003948032

Rasl12	RAS-like, family 12	1,542	0.0016527065
Smtnl1	smoothelin-like 1	1,542	0.0025668738
Gm5595	predicted gene 5595	1,537	0.002425921
Pbx4	pre B cell leukemia homeobox 4	1,537	0.0018666127
Etv3	ets variant 3	1,532	0.002837071
Fam49a	family with sequence similarity 49, member A	1,532	0.0028897594
Msmp	microseminoprotein, prostate associated	1,532	0.0013523372
Cdon	cell adhesion molecule-related/down-regulated by oncogenes	1,526	0.0024592453
Atp13a5	ATPase type 13A5	1,521	0.002064307
Inmt	indolethylamine N-methyltransferase	1,521	0.0027361973
Gm1818	predicted gene 1818 [Source:MGI Symbol;Acc:MGI:3037676]	1,516	0.0032720887
Nupl2	nucleoporin like 2	1,516	0.004172296
Pgr15l	G protein-coupled receptor 15-like	1,516	0.0024200666
Trp53i11	transformation related protein 53 inducible protein 11	1,516	0.004660002
Aldh2	aldehyde dehydrogenase 2, mitochondrial	1,510	0.0034625777
Txnrd3	thioredoxin reductase 3	1,510	0.0027334956
Aim2	absent in melanoma 2	1,510	0.002627218
Fhit	fragile histidine triad gene	1,510	0.0038422048
Entpd5	ectonucleoside triphosphate diphosphohydrolase 5	1,505	0.0027798794
Gjb4	gap junction protein, beta 4	1,505	0.0046388363
Ndufa4l2	NADH dehydrogenase (ubiquinone) 1 alpha subcomplex, 4-like 2	1,505	0.0017959111
Olfr822	olfactory receptor 822	1,505	0.0020602539
Pou4f1	POU domain, class 4, transcription factor 1	1,500	0.004528956
Syt13	synaptotagmin XIII	1,500	0.0029388454
Dpep1	dipeptidase 1 (renal)	1,495	0.0030883546
Olfr1258	olfactory receptor 1258	1,495	0.0026402774
Fbln5	fibulin 5	1,490	0.0046690083
Krt14	keratin 14	1,490	0.002484914
Olfr452	olfactory receptor 452	1,490	0.003948032
Tuft1	tuftelin 1	1,490	0.0048991265
Ncoa7	nuclear receptor coactivator 7	1,485	0.003563001
Nipal3	NIPA-like domain containing 3	1,485	0.004543367
Mest	mesoderm specific transcript	1,479	0.0041196076
Mtfr2	mitochondrial fission regulator 2	1,474	0.0044375393
Ssx2ip	synovial sarcoma, X breakpoint 2 interacting protein	1,474	0.0041115014
Stard3	START domain containing 3	1,474	0.0044524004
Wasf1	WAS protein family, member 1	1,474	0.0032923534
Ica1	islet cell autoantigen 1	1,469	0.0048032063
Nmrk1	nicotinamide riboside kinase 1	1,469	0.0043871026
Sord	sorbitol dehydrogenase	1,469	0.0047469153
Kdm1b	lysine (K)-specific demethylase 1B	1,464	0.0040673693
Slx4ip	SLX4 interacting protein	1,464	0.0049171397
Klra17	killer cell lectin-like receptor, subfamily A, member 17	1,459	0.0037521392
Smr2	submaxillary gland androgen regulated protein 2	1,459	0.0045280554
Pigw	phosphatidylinositol glycan anchor biosynthesis, class W	1,454	0.0044258307
Ano2	anoctamin 2	1,444	0.0044839233
Klk1b11	kallikrein 1-related peptidase b11	1,444	0.004620823
Ppp1r3f	protein phosphatase 1, regulatory (inhibitor) subunit 3F	1,444	0.0031455462
1700011H14Rik	RIKEN cDNA 1700011H14 gene	1,439	0.004207872
